# ICP0 antagonizes Stat 1-dependent repression of herpes simplex virus: implications for the regulation of viral latency

**DOI:** 10.1186/1743-422X-3-44

**Published:** 2006-06-09

**Authors:** William P Halford, Carla Weisend, Jennifer Grace, Mark Soboleski, Daniel JJ Carr, John W Balliet, Yumi Imai, Todd P Margolis, Bryan M Gebhardt

**Affiliations:** 1 Dept of Veterinary Molecular Biology, Montana State University, Bozeman, MT, USA; 2 Dept of Microbiology and Immunology, Tulane University Medical School, New Orleans, LA, USA; 3 Dean McGee Eye Institute, University of Oklahoma Health Sciences Center, Oklahoma City, OK, USA; 4 Beth Israel Deaconess Medical Center, Harvard Medical School, Boston, MA, USA; 5 Francis I. Proctor Foundation, University of California, San Francisco, CA, USA; 6 Dept of Ophthalmology, Louisiana State University Health Sciences Center, New Orleans, LA, USA

## Abstract

**Background:**

The herpes simplex virus type 1 (HSV-1) ICP0 protein is an E3 ubiquitin ligase, which is encoded within the HSV-1 latency-associated locus. When ICP0 is not synthesized, the HSV-1 genome is acutely susceptible to cellular repression. Reciprocally, when ICP0 is synthesized, viral replication is efficiently initiated from virions or latent HSV-1 genomes. The current study was initiated to determine if ICP0's putative role as a viral interferon (IFN) antagonist may be relevant to the process by which ICP0 influences the balance between productive replication versus cellular repression of HSV-1.

**Results:**

Wild-type (ICP0^+^) strains of HSV-1 produced lethal infections in *scid *or *rag2*^-/- ^mice. The replication of ICP0^- ^null viruses was rapidly repressed by the innate host response of *scid *or *rag2*^-/- ^mice, and the infected animals remained healthy for months. In contrast, *rag2*^-/- ^mice that lacked the IFN-α/β receptor (*rag2*^-/- ^*ifnar*^-/-^) or Stat 1 (*rag2*^-/- ^*stat1*^-/-^) failed to repress ICP0^- ^viral replication, resulting in uncontrolled viral spread and death. Thus, the replication of ICP0^- ^viruses is potently repressed *in vivo *by an innate immune response that is dependent on the IFN-α/β receptor and the downstream transcription factor, Stat 1.

**Conclusion:**

ICP0's function as a viral IFN antagonist is necessary *in vivo *to prevent an innate, Stat 1-dependent host response from rapidly repressing productive HSV-1 replication. This antagonistic relationship between ICP0 and the host IFN response may be relevant in regulating whether the HSV-1 genome is expressed, or silenced, in virus-infected cells *in vivo*. These results may also be clinically relevant. IFN-sensitive ICP0^- ^viruses are avirulent, establish long-term latent infections, and induce an adaptive immune response that is highly protective against lethal challenge with HSV-1. Therefore, ICP0^- ^viruses appear to possess the desired safety and efficacy profile of a live vaccine against herpetic disease.

## Background

Herpesviruses are double-stranded DNA viruses that establish life-long infections in their animal hosts, and which alternate between two programs of gene expression: ***i***. productive replication, or ***ii***. latent infection in which most of the viral genome is transcriptionally silent.

**Figure 1:**
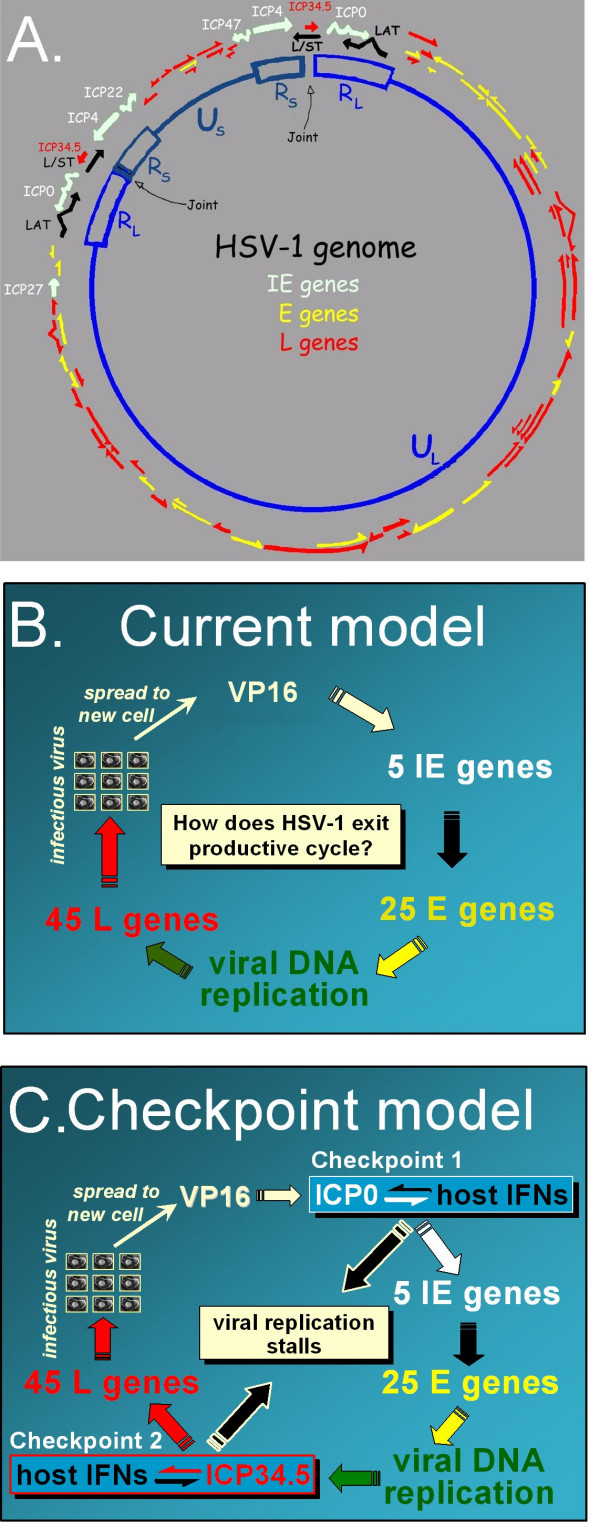
**Two alternative models of HSV-1 gene regulation**. **A**. Genetic organization of the HSV-1 genome. The long-repeated (R_L_) and short-repeated (R_S_) regions of the HSV-1 genome regulate expression of 4 of 5 immediate-early (IE) genes (white arrows). The unique long (U_L_) and unique short (U_S_) regions contain most of the early (E) and late (L) genes (yellow and red arrows). The 15 kb R_L _and R_S _regions include a 2 kb recombinogenic 'joint' sequence, the ICP34.5 gene (red arrow), and the LAT and L/ST genes which are repressed during productive replication (black arrows). **B**. The current model of HSV-1 gene regulation [1] describes a cascade of IE → E → L gene expression. **C**. The proposed Checkpoint model predicts that HSV-1 gene expression proceeds by the accepted cascade, but that viral gene expression can be blocked during viral IE mRNA synthesis if ICP0 is not synthesized (Checkpoint 1) or can be blocked during viral L protein synthesis if ICP34.5 is not synthesized (Checkpoint 2).

Herpes simplex virus 1 (HSV-1) and 2 (HSV-2) are the human herpesviruses that cause recurrent cold sores and genital herpes. The regulation of gene expression from the co-linear HSV-1 and HSV-2 genomes has been described in terms of a cascade of expression of immediate-early (IE), early (E), and late (L) genes (Fig. 1A). This model was proposed 30 years ago to describe HSV-1 gene expression in cultured cells [1]. The model predicts that HSV-1 infection of a cell always leads to the production of infectious viral progeny (Fig. 1B). The model is accurate for wild-type HSV-1 *in vitro*, but fails to account for the most defining feature of HSV-1 and HSV-2: their capacity to establish latent infections *in vivo*.

The long-repeated, R_L_, regions of the HSV-1 genome appear to regulate HSV-1's capacity to alternate between two programs of gene expression: productive replication or non-productive infection (Fig. 1A). Each copy of the R_L _region encodes the latency-associated transcript (LAT), the long-short spanning transcript (L/ST), infected cell protein 0 (ICP0), and infected cell protein 34.5 (ICP34.5) (Fig. 1A). Both the LAT and L/ST genes produce RNA transcripts that encode no known protein [2,3]. A viral protein, ICP4, blocks the transcription of the LAT and L/ST genes during productive replication by binding the 5' end of each gene [3,4]. The ICP0 and ICP34.5 genes, which lie on the opposite strand of DNA, promote HSV-1 replication. ICP0 is an E3 ubiquitin ligase that overcomes cellular repression of HSV-1 [5,6]. ICP34.5 antagonizes protein kinase R (PKR)-induced shutoff of viral protein translation by inducing the dephosphorylation of the translation initiation factor, eIF-2α [7,8].

The current model of HSV-1 gene regulation ascribes no significance to the genes in the HSV-1 latency-associated locus, and fails to explain why both R_L_-encoded proteins function as viral interferon (IFN) antagonists [9-11]. When IFNs bind their cognate receptors at the cell surface, the signal transducer and activator of transcription 1 (Stat 1) protein is phosphorylated and acts in concert with other transcription factors to induce IFN-stimulated gene expression, thus creating an antiviral state in the host cell [12,13]. Wild-type HSV-1 is remarkably resistant to the antiviral state induced by activation of either IFN-α/β receptors ***or ***IFN-γ receptors [14]. In contrast, HSV-1 ICP0^- ^or ICP34.5^- ^mutants are hypersensitive to the antiviral state induced by activation of IFN-α/β receptors *in vitro *[14-16]. Reciprocally, ICP0^- ^and ICP34.5^- ^viruses exhibit improved replication in IFN-α/β receptor-knockout mice [11,17].

The opposing forces produced by IFN-inducible cellular repressors and the R_L_-encoded viral IFN antagonists, ICP0 and ICP34.5, may form two checkpoints that regulate whether or not HSV-1 completes its replication cycle in an infected cell *in vivo*. This hypothesis can be integrated into the current model of HSV-1 gene regulation via two modifications (Fig. 1C):

**1**. ICP0 and IFN-inducible cellular repressor(s) form an ON-OFF switch that controls whether or not viral IE mRNA synthesis occurs in an infected cell (Checkpoint 1).

**2**. ICP34.5 and an IFN-inducible cellular repressor, PKR, form an ON-OFF switch that controls whether or not viral L protein synthesis occurs in an infected cell (Checkpoint 2).

The OFF event at Checkpoint 1 is predicted to occur when ICP0 is not synthesized, and the host IFN response stably represses viral IE mRNA synthesis [14,15]. The OFF event at Checkpoint 2 is predicted to occur when ICP34.5 is not synthesized, and the host IFN response acts through PKR to induce the shutoff of viral L protein synthesis [11,18].

The proposed Checkpoint Model represents an attempt to explain how the genes in the HSV-1 latency-associated locus may influence the decision-making process that dictates whether the HSV-1 genome is *expressed *(productive replication) or *repressed *(quiescent infection) when HSV-1 enters a cell *in vivo*. The evidence that supports the model is circumstantial, and thus the accuracy of the model is questionable. For example, the Checkpoint Model assumes that the primary function of ICP0 lies in antagonizing IFN-inducible repression of HSV-1 *in vivo *(Fig. 1C). Several observations are consistent with, but do not prove, this hypothesis [14,15,17]. The current study was initiated to test two key predictions of the Checkpoint Model: ***i***. ICP0^- ^mutants should be susceptible to repression by the innate immune response *in vivo*, and ***ii***. ICP0^- ^mutants should replicate efficiently and be fully virulent in hosts that are IFN-unresponsive.

Given the extensive literature on HSV-1, no one manuscript can satisfactorily *prove *the accuracy of a new *in vivo *paradigm of HSV-1 gene regulation. On the other hand, the need for an improved *in vivo *model is clear. A model is needed which identifies the host and/or viral factors that can influence whether HSV-1 infection of a cell leads to productive replication or non-productive infection *in vivo*. The goals of the current study are to introduce the possibility that the cessation of HSV-1 replication *in vivo *may be regulated by an equilibrium between the host IFN response and viral IFN antagonists. The *in vivo *behavior of HSV-1 ICP0^- ^mutants is described, which is inexplicable in terms of the current model of HSV-1 gene regulation, but which logically follows from the proposed Checkpoint Model (Fig. 1C). The evidence that supports this newly proposed model is discussed.

## Results

### Failure to express ICP0 allows HSV-1 to be stably repressed in *scid* mice

**Figure 2:**
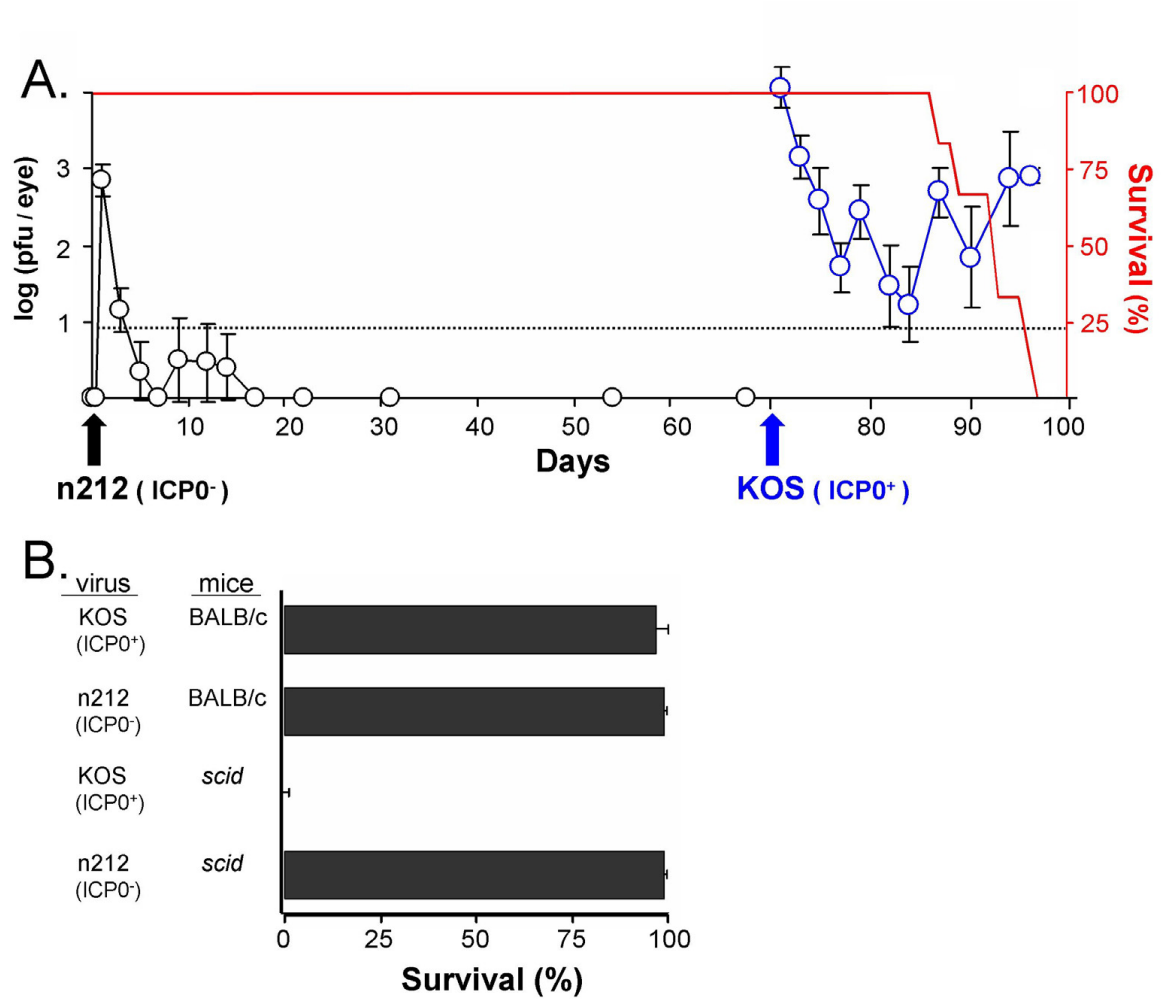
**An ICP0^- ^virus is avirulent in *scid *mice**. **A**. *Scid *mice were inoculated with 2 × 10^5 ^pfu per eye of the ICP0^- ^virus n212 (n = 6 mice). The mean ± sem of the logarithm of viral titers recovered from mouse eyes is plotted over time (open black symbols). The survival of n212-infected *scid *mice is plotted over time (red line). On day 70 p.i., n212-infected *scid *mice were challenged with 2 × 10^5 ^pfu per eye of wild-type HSV-1 strain KOS (subsequent viral titers are shown as open blue symbols). The dashed line indicates the lower limit of detection of the plaque assay used to determine viral titers. **B**. Survival of BALB/c mice versus *scid *mice infected with KOS or n212. Bars represent the mean ± sem of survival frequency of ICP0^- ^virus-infected mice at day 60 p.i. (n = 5 experiments; Σn = 30 mice per group).

BALB/c severe-combined immunodeficient (*scid*) mice were inoculated with 2 × 10^5 ^pfu per eye of an HSV-1 ICP0^- ^virus, n212 (described in Table 1). At 2 and 12 hours post inoculation (p.i.), infectious virus was not detectable in the ocular tear film of mice. At 24 hours p.i., an average of 3000 pfu of n212 was recovered from the eyes of *scid *mice (black circles in Fig. 2A). Replication of the ICP0^- ^virus remained low to undetectable between days 3 and 70 p.i., and the n212 infection produced no disease. Thus, 100% of n212-infected *scid *mice remained healthy and survived for 70 days p.i. (red line in Fig. 2A). Secondary challenge with wild-type HSV-1 strain KOS on day 70 p.i. verified that n212-infected *scid *mice had not mounted an adaptive immune response to HSV-1. KOS sustained high levels of replication in the eyes of *scid *mice, and produced a uniformly lethal infection (Fig. 2A).

**Table 1:** Viruses and mice used in this study.

**Genetic Background**	**Virus**	**Genotype of virus**	**Phenotype of virus**
	KOS	wild-type	wild-type
	KOS-GFP^a^	CMV-GFP cassette between UL26 and UL27 genes	wild-type [56]
KOS	n212^b^	ICP0^- ^null	IFN-sensitive [14]
	0^-^-GFP^c^	ICP0^- ^null	IFN-sensitive (Fig. 5B)
	n12^d^	ICP4^- ^null	replication-defective [55]

**Genetic Background**	**Mouse**	**Immunological status of mouse**	

BALB/c	BALB/c *scid*^e^	immunocompetent lymphocyte-deficient [64]	

	Strain 129	immunocompetent	
	PML^-/- f^	immunocompetent [63]	
	*rag2*^-/- g^	lymphocyte-deficient [64]	
	*ifngr*^-/-^	IFN-γ receptor-null [65]	
	*ifnar*^-/-^	IFN-α/β receptor-null [66]	
Strain 129	*ifnar*^-/- ^*ifngr*^-/-^	IFN-α/β receptor-null + IFN-γ receptor-null [67]	
	*stat1*^-/-^	Stat 1-null [68]	
	*rag2*^-/- ^*stat1*^-/-^	lymphocyte-deficient + Stat 1-null	
	*rag2*^-/- ^*ifnar*^-/-^	lymphocyte-deficient + IFN-α/β receptor-null	

^a ^The HSV-1 recombinant virus KOS-GFP contains a 2.0 kbp insertion in the intergenic region between the UL26 and UL27 genes of HSV-1 strain KOS, which contains a cytomegalovirus (CMV) IE promoter driving the expression of the green-fluorescent protein (GFP).

^b ^The ICP0^- ^null mutant n212 contains a 14 bp insertion, ctagactagtctag, in codon 212 of the ICP0 gene of HSV-1 strain KOS, which inserts stop codons into all three open-reading frames of the ICP0-encoding DNA strand. Illustrated in Figure 4.

^c ^The ICP0^- ^null mutant 0^-^-GFP contains an ~770 bp insertion in codon 105 of the ICP0 gene of HSV-1 strain KOS, which inserts a GFP coding sequence and 'taa' terminator codon into the ICP0 open-reading frame. Illustrated in Figure 4.

^d ^The ICP4^- ^null mutant n12 contains a 16 bp insertion, ggctagttaactagcc, in codon 262 of the ICP4 gene of HSV-1 strain KOS, which inserts stop codons into all three open-reading frames of the ICP4-encoding DNA strand.

^e ^SCID: severe-combined immunodeficiency is a phenotype that results from any one of dozens of genetic mutations that block lymphocyte maturation. The genetic lesion that accounts for the SCID phenotype of *scid *mice lies in the gene that encodes the catalytic subunit of the DNA-dependent protein kinase. This protein is necessary to repair double-stranded DNA breaks, and is essential to complete V-D-J recombination of either the T cell receptor gene or the B cell receptor gene.

^f ^PML: a protooncogene which, when mutated, is associated with promyelocytic leukemia.

^g ^RAG2: recombination-activated gene 2, which encodes a protein necessary to initiate V-D-J recombination of the T cell receptor gene or B cell receptor gene.

Multiple experiments confirmed that the ICP0^- ^virus n212 was avirulent in *scid *mice, whereas the wild-type KOS strain produced uniformly lethal infections in *scid *mice (Fig. 2B). Moreover, n212 appeared to rapidly exit the productive cycle of viral replication in *scid *mice based on ***i***. low to undetectable levels of infectious virus in the tear film of *scid *mice between 3 and 70 days p.i., ***ii***. undetectable levels of infectious virus in homogenates of eyes or trigeminal ganglia (TG) at 35 or 70 days p.i. (n = 10 tissues per time point), and ***iii***. the fact that n212-infected *scid *mice remained indistinguishable from uninfected mice for more than 2 months p.i.

### The *in vivo* repression of ICP0^- ^viruses is Stat 1-dependent

**Figure 3:**
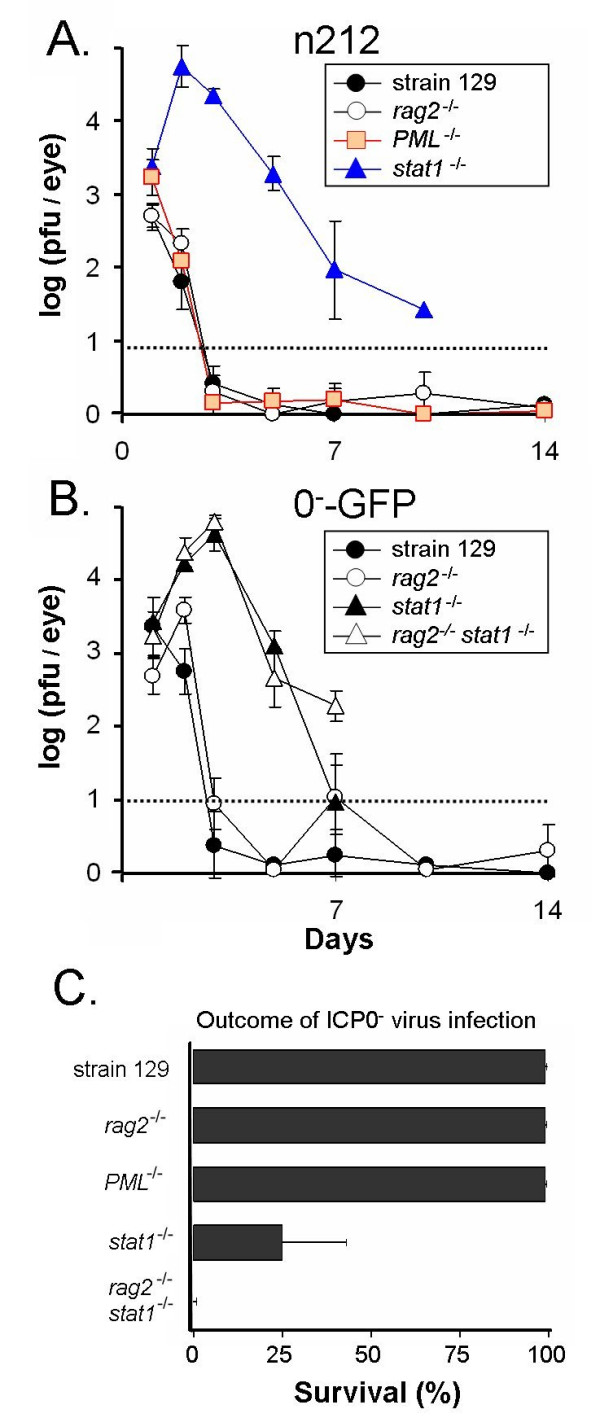
**Loss of Stat 1 alleviates innate host repression of ICP0^- ^viruses *in vivo***. **A**. Strain 129 mice, *rag2*^-/- ^mice, *PML*^-/- ^mice, or *stat1*^-/- ^mice were inoculated with 2 × 10^5 ^pfu per eye of the ICP0^- ^virus n212 (n = 4 mice per group). The mean ± sem of the logarithm of viral titers recovered from mouse eyes is plotted over time. **B**. Strain 129 mice, *rag2*^-/- ^mice, *stat1*^-/- ^mice, or *rag2*^-/- ^*stat1*^-/- ^mice were inoculated with 2 × 10^5 ^pfu per eye of the ICP0^- ^virus, 0^-^-GFP (n = 4 mice per group). Dashed lines indicate the lower limit of detection of the plaque assay. **C**. Survival of strain 129 mice, *rag2*^-/- ^mice, *PML*^-/- ^mice, *stat1*^-/- ^mice, or *rag2*^-/- ^*stat1*^-/- ^mice infected with the ICP0^- ^viruses, n212 or 0^-^-GFP. Bars represent the mean ± sem of survival frequency of ICP0^- ^virus-infected mice at day 60 p.i. (n = 3 experiments; Σn = 14 mice per group).

To determine if the IFN-induced antiviral state [19,20] or the IFN-induced pro-myelocytic leukemia (PML)-associated protein [21] is relevant to the innate mechanisms by which mice rapidly repress HSV-1 ICP0^- ^null mutants *in vivo*, the acute ocular replication of an ICP0^- ^virus was compared in wild-type strain 129 mice, recombination-activated gene 2^-/- ^(*rag2*^-/-^) mice, PML^-/- ^mice, or *stat1*^-/- ^mice (described in Table 1). Following inoculation with 2 × 10^5 ^pfu per eye of the HSV-1 ICP0^- ^virus n212, ~1000 pfu per eye of virus was recovered from all strains of mice at 24 hours p.i. (Fig. 3A). On day 3 p.i., titers of infectious n212 were ~1000-fold higher in the eyes of *stat1*^-/- ^mice relative to the other strains of mice (Fig. 3A). While *rag2*^-/- ^mice survived n212 infection for >60 days, n212 infection was lethal in 3 of 4 *stat1*^-/- ^mice by day 12 p.i. Loss of Stat 1 alleviated host repression of n212, but loss of PML did not produce a comparable effect. *PML*^-/- ^mice repressed n212 replication at the site of ocular inoculation with the same kinetics as wild-type mice and *rag2*^-/- ^mice (Fig. 3A).

**Figure 4:**
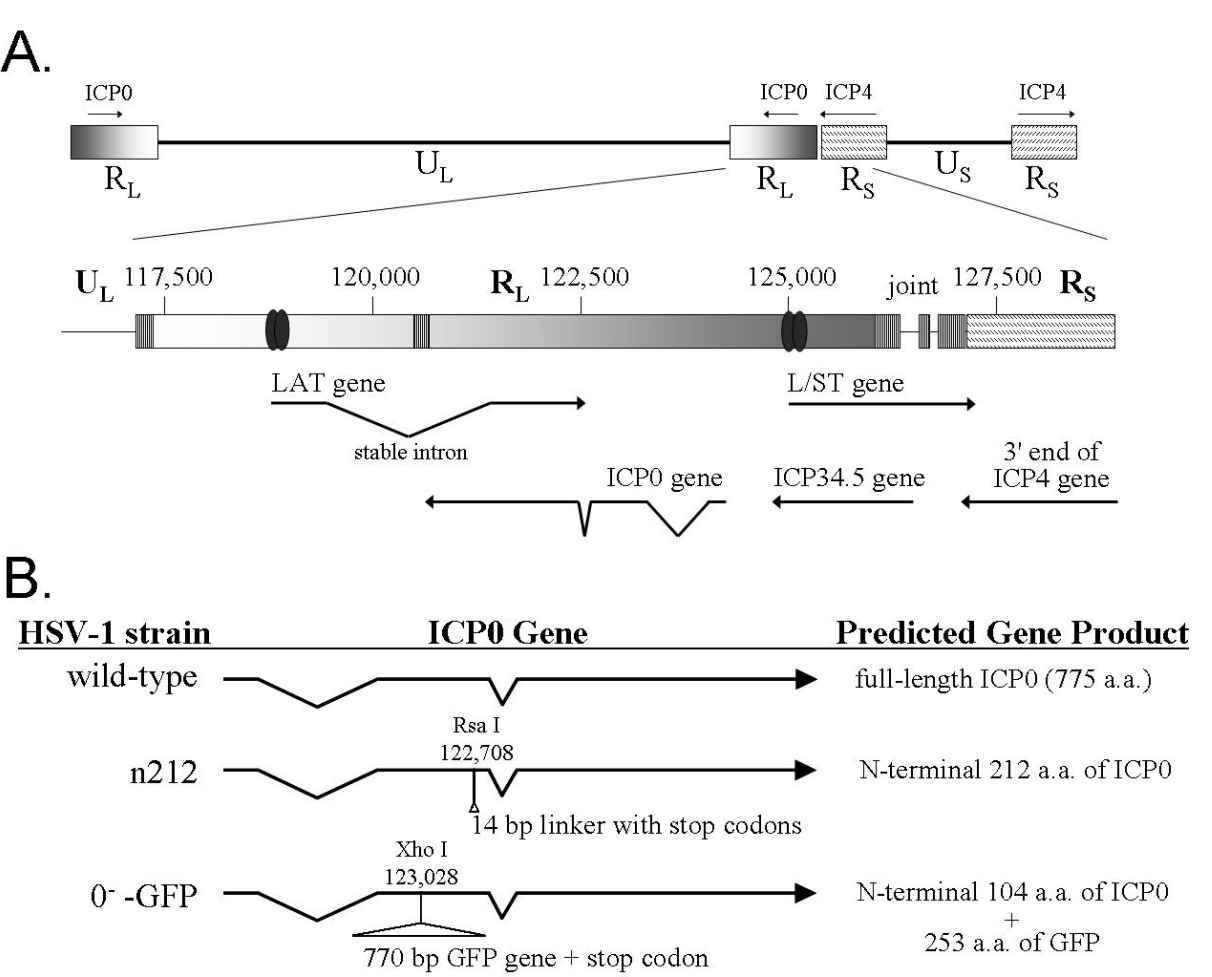
**The R_L _region**. **A**. Genetic organization of the HSV-1 R_L _region. Numbers refer to base positions in the prototype HSV-1 genome, and arrows denote the LAT, L/ST, ICP34.5, and ICP0 primary transcripts. Reiterated DNA sequences in the R_L _region are denoted by small boxes containing vertical bars. The location of the DNA sequences to which ICP4 homodimers bind in the LAT and L/ST genes is denoted by pairs of black ovals at the 5' end of each gene. **B**. The ICP0 genes of wild-type HSV-1 and the ICP0^- ^viruses n212 and 0^-^-GFP. The mutation in n212 introduces a 14 bp linker sequence into codon 212 of the ICP0 open-reading frame, which terminates protein translation [53]. The insertion mutation in 0^-^-GFP introduces an ~770 bp green-fluorescent protein (GFP) coding sequence in-frame with the ICP0 gene. The resulting mRNA is predicted to encode the N-terminal 104 amino acids of ICP0 fused to a 14 amino acid linker and 239 amino acids of C-terminal GFP.

The n212 virus bears a small 14 bp linker insertion in the ICP0 gene (Fig. 4), which can revert to a wild-type ICP0 gene by excision of the linker sequence *in vivo *(unpublished observation). To verify that an ICP0^- ^virus itself, as opposed to a wild-type revertant, was capable of producing disease in *stat1*^-/- ^mice, a second experiment was performed with the ICP0^- ^virus, 0^-^-GFP (Fig. 4; described in Table 1). At 24 hours p.i., ~1000 pfu per eye of 0^-^-GFP was recovered from the eyes of wild-type mice, *rag2*^-/- ^mice, *stat1*^-/- ^mice, or *rag2*^-/- ^*stat1*^-/- ^mice (Fig. 3B). On day 3 p.i., titers of infectious 0^-^-GFP were ~1000-fold higher in the eyes of *stat1*^-/- ^mice and *rag2*^-/- ^*stat1*^-/- ^mice relative to wild-type mice or *rag2*^-/- ^mice (Fig. 3B). At all times p.i., the virus recovered from *stat1*^-/- ^mice and *rag2*^-/- ^*stat1*^-/- ^mice retained the GFP insertion in the ICP0 gene (Fig. 4) based on the GFP^+ ^phenotype of plaques that formed in plaque assays. In this experiment, 0^-^-GFP infection was lethal in 100% of *stat1*^-/- ^mice and *rag2*^-/- ^*stat1*^-/- ^mice by day 12 p.i.

In multiple experiments, the ICP0^- ^viruses n212 and 0^-^-GFP did not produce disease in strain 129 mice, *rag2*^-/- ^mice, and *PML*^-/- ^mice, and 100% of the mice survived for 60 days p.i. (Fig. 3C). In contrast, n212 and 0^-^-GFP produced lethal infections in 50 to 100% of *stat1*^-/- ^mice and in 100% of *rag2*^-/- ^*stat1*^-/- ^mice (Fig. 3C). Thus, the IFN-activated Stat 1 transcription factor was required for *rag2*^-/- ^mice to rapidly repress the replication of ICP0^- ^viruses *in vivo*.

### *Rag2^-/-^stat 1^-/-^* mice die of uncontrolled viral spread, not human handling

**Figure 5:**
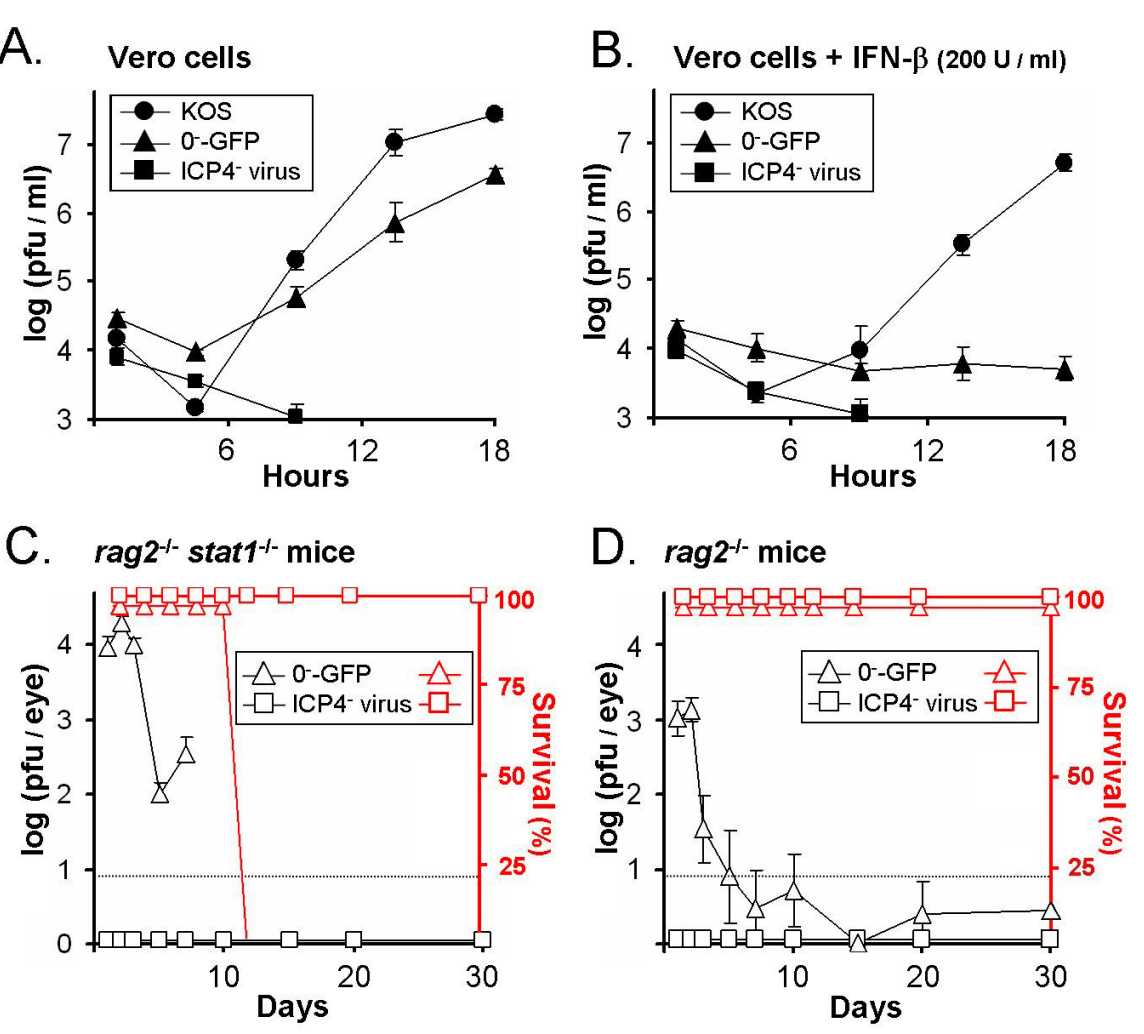
**Replication of ICP0^- ^and ICP4^- ^viruses in cell culture and immunodeficient mice**. Vero cells were **A**. untreated or **B**. treated with 200 U per ml of IFN-β and were inoculated with 2.5 pfu per cell of wild-type HSV-1 (KOS), an ICP0^- ^virus (0^-^-GFP), or an ICP4^- ^virus (n12). The mean ± sem of the logarithm of viral titers recovered from Vero cells is plotted over time (n = 4 per time point). **C**. *Rag2*^-/- ^*stat1*^-/- ^mice and **D**. *rag2*^-/- ^mice were inoculated with 2 × 10^5 ^pfu per eye of the ICP0^- ^virus 0^-^-GFP or the ICP4^- ^virus n12 (n = 4 mice per group). The mean ± sem of the logarithm of viral titers recovered from mouse eyes is plotted over time (open black symbols). Dashed lines indicate the lower limit of detection of each plaque assay. The survival of 0^-^-GFP-infected mice and ICP4^- ^virus-infected mice is plotted over time (open red symbols).

Given the severity of the immunodeficiency of *rag2*^-/- ^*stat1*^-/- ^mice, mortality in this strain may have been due to secondary infections introduced by corneal scarification and/or human handling. To address this possibility, a series of *in vitro *and *in vivo *experiments were performed comparing the ICP0^- ^virus, 0^-^-GFP, to the replication-defective HSV-1 ICP4^- ^virus, n12 (described in Table 1). *In vitro*, an inoculum of 2.5 pfu per cell of 0^-^-GFP replicated relatively efficiently in Vero cells, whereas the ICP4^- ^virus produced no viral progeny (Fig. 5A). When Vero cells were treated with the IFN-α/β receptor agonist, IFN-β, both 0^-^-GFP and the ICP4^- ^virus failed to produce viral progeny (Fig. 5B). In contrast, wild-type HSV-1 resisted repression by IFN-β and was only transiently delayed in its replication relative to untreated cells (Fig. 5B). Thus, ICP0 was required for HSV-1 replication when cultured cells were exposed to the Stat 1 activator, IFN-β.

*In vivo*, 0^-^-GFP replicated to high titers in the eyes of *rag2*^-/- ^*stat1*^-/- ^mice, acute swelling of periocular tissue occurred, and none of the mice survived beyond day 11 p.i. (Fig. 5C). In contrast, the ICP4^- ^virus failed to replicate in *rag2*^-/- ^*stat1*^-/- ^mice or *rag2*^-/- ^mice, and all of the ICP4^- ^virus-infected mice remained healthy for the 30-day test period (Fig. 5C and 5D). In *rag2*^-/- ^mice, which retained a functional Stat 1 pathway, the ocular replication of 0^-^-GFP was rapidly repressed and 100% of 0^-^-GFP-infected *rag2*^-/- ^mice remained healthy for the 30-day observation period (Fig. 5D). Thus, the pathogenesis of 0^-^-GFP infection observed in *rag2*^-/- ^*stat1*^-/- ^mice appeared to be the result of unchecked viral replication, and was not the result of an unanticipated infection with the flora of the mice or their human handlers.

### Stat 1 is necessary to restrict wild-type HSV-1 spread *in vivo*

**Figure 6:**
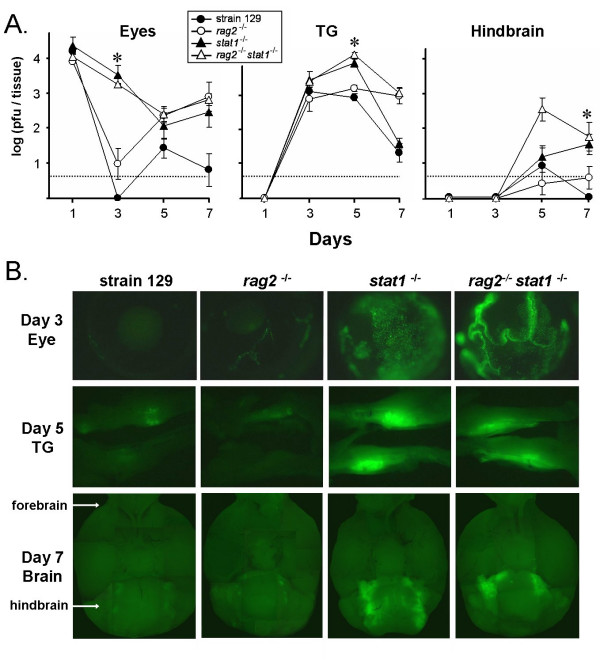
**A Stat1-dependent host response restricts the spread of HSV-1 strain KOS-GFP into the central nervous system**. Strain 129 mice, *rag2*^-/- ^mice, *stat1*^-/- ^mice, or *rag2*^-/- ^*stat1*^-/- ^mice were inoculated with 2 × 10^5 ^pfu per eye of HSV-1 strain KOS-GFP. **A**. The mean ± sem of the logarithm of viral titers recovered from homogenates of mouse eyes, TG, and hindbrain is plotted as a function of the time p.i. at which tissues were harvested (n = 5 per time point). Asterisks denote significant differences between *stat1*^+/+ ^versus *stat1*^-/- ^tissues (p < 0.001, as determined by two-way ANOVA). Dashed lines indicate the lower limit of detection of each plaque assay. **B**. GFP expression in tissues of KOS-GFP-infected mice. Representative photographs are shown of eyes harvested on day 3 p.i. (4× magnification, 250 ms exposure), TG harvested on day 5 p.i. (2× magnification, 500 ms exposure), and the ventral side of brains harvested on day 7 p.i. (2× magnification, 1000 ms exposure).

To determine if the Stat 1-dependent host response was relevant to wild-type HSV-1 infection, strain 129 mice, *rag2*^-/- ^mice, *stat1*^-/- ^mice, or *rag2*^-/- ^*stat1*^-/- ^mice were inoculated with 2 × 10^5 ^pfu per eye of KOS-GFP, a GFP-expressing recombinant of strain KOS (described in Table 1). On day 1 p.i., titers of KOS-GFP were equivalent in the eyes of all groups of mice (Fig. 6A). On day 3 p.i., KOS-GFP titers were ~100-fold greater in the eyes of *stat1*^-/- ^and *rag2*^-/- ^*stat1*^-/- ^mice relative to wild-type and *rag2*^-/- ^mice (Fig. 6A). Likewise, GFP fluorescence was nearly undetectable in the eyes of wild-type and *rag2*^-/- ^mice on day 3 p.i., but persisted in the eyes of *stat1*^-/- ^mice and *rag2*^-/- ^*stat1*^-/- ^mice (Fig. 6B). Infectious KOS-GFP titers were ~10-fold higher on day 5 p.i. in the TG of *stat1*^-/- ^and *rag2*^-/- ^*stat1*^-/- ^mice relative to wild-type and *rag2*^-/- ^mice (Fig. 6A). Likewise, GFP fluorescence emanated from large tracts of cells in the TG of *stat1*^-/- ^and *rag2*^-/- ^*stat1*^-/- ^mice on day 5 p.i., whereas the TG of wild-type and *rag2*^-/- ^mice possessed discrete foci of GFP fluorescence (Fig. 6B). In wild-type and *rag2*^-/- ^mice, only limited spread of KOS-GFP to the hindbrain of mice was observed on days 5 and 7 p.i. (Fig. 6A, 6B). In contrast, GFP expression was evident in the hindbrain of 17 of 20 *stat1*^-/- ^and *rag2*^-/- ^*stat1*^-/- ^mice on days 5 and 7 p.i. (Fig. 6B). Thus, an innate Stat 1-dependent host response is necessary to prevent extensive spread of KOS-GFP infection from the corneal epithelium to the central nervous system of mice.

### IFN receptors are integral to the innate response that limits HSV-1 spread *in vivo*

**Figure 7:**
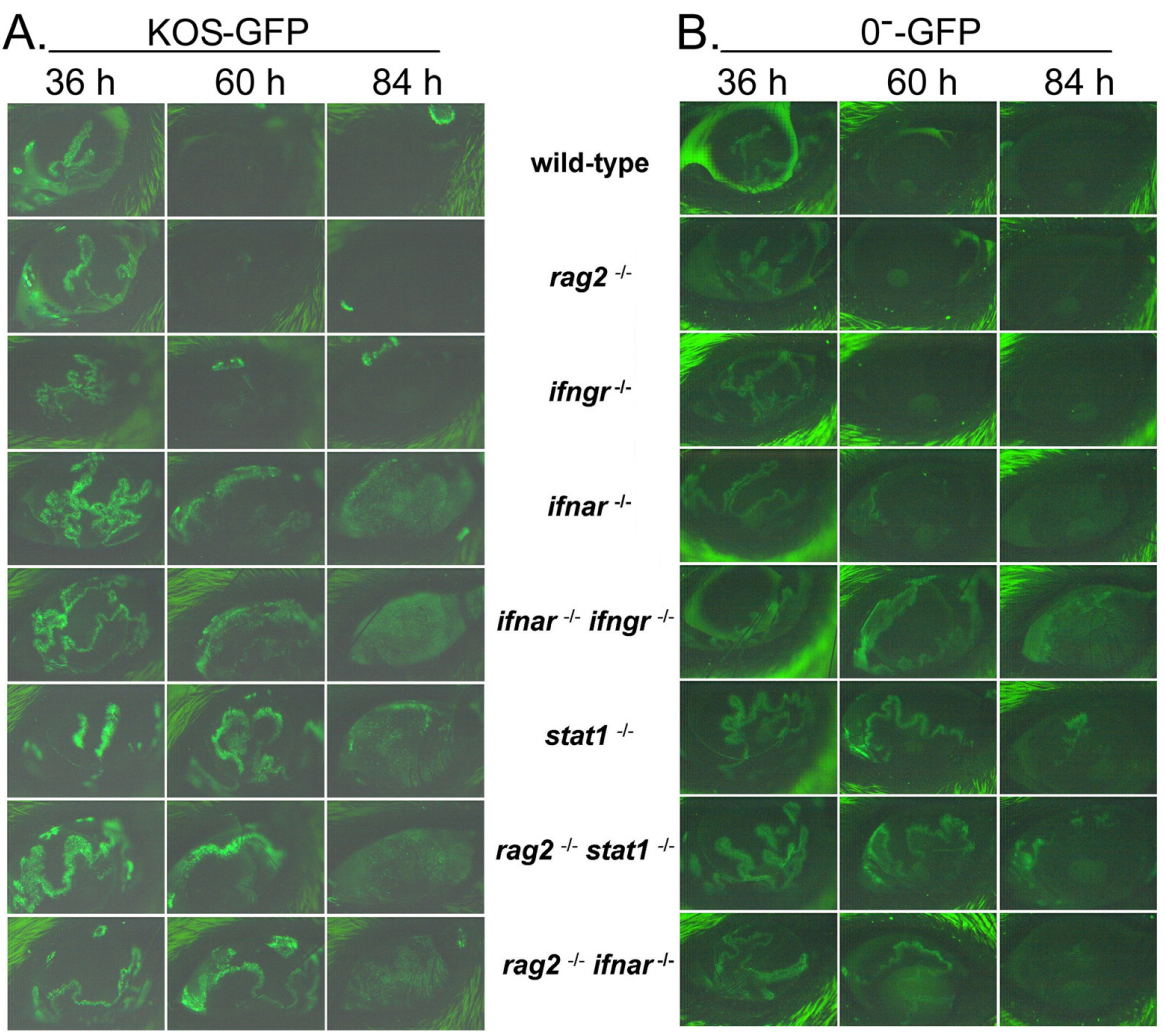
**Loss of IFN-α/β receptors or Stat 1 impairs an innate host response that represses KOS-GFP and 0^-^-GFP at the site of inoculation**. Mice were inoculated with 2 × 10^5 ^pfu per eye of **A**. HSV-1 strain KOS-GFP, or **B**. the ICP0^- ^virus, 0^-^-GFP. GFP fluorescence was recorded in the right eyes of strain 129 mice, *rag2*^-/- ^mice (lymphocyte-deficient), *ifngr*^-/- ^mice (IFN-γ receptor-null), *ifnar*^-/- ^mice (IFN-α/β receptor-null), *ifnar*^-/- ^*ifngr*^-/- ^mice, *stat1*^-/- ^mice, *rag2*^-/- ^*stat1*^-/- ^mice, and *rag2*^-/- ^*ifnar*^-/- ^mice. Representative photographs are shown of GFP fluorescence in the virus-infected eye of one mouse per group photographed over time at 36, 60, and 84 hours p.i. (4× magnification; 39 ms exposure for KOS-GFP; 63 ms exposure for 0^-^-GFP).

To determine if host IFNs are the principal activators of Stat 1-dependent repression of HSV-1, the progression of KOS-GFP (ICP0^+^) or 0^-^-GFP (ICP0^-^) infection was compared in mice of the following genotypes: **1**. wild-type, **2**. *rag2*^-/-^, **3**. *ifngr*^-/- ^(IFN-γ receptor-null), **4**. *ifnar*^-/- ^(IFN-α/β receptor-null), **5**. *ifnar*^-/- ^*ifngr*^-/-^, **6**. *stat1*^-/-^, **7**. *rag2*^-/- ^*stat1*^-/-^, or **8**. *rag2*^-/- ^*ifnar*^-/- ^(described in Table 1). Following inoculation with 2 × 10^5 ^pfu per eye of KOS-GFP, similar levels of GFP fluorescence were observed in the corneas of mice at 36 hours p.i. (Fig. 7A). By 60 and 84 hours p.i., GFP fluorescence was nearly undetectable in the corneas of wild-type, *rag2*^-/-^, and *ifngr*^-/- ^mice (Fig. 7A). All strains of mice with a defect in the *ifnar *or *stat1 *genes failed to limit KOS-GFP spread, and thus GFP fluorescence was still evident throughout the cornea at 84 hours p.i. (Fig. 7A). Likewise, KOS-GFP titers were an average 10- to 300-times higher in the tear film of *ifnar*^-/-^, *ifnar*^-/- ^*ifngr*^-/-^, *stat1*^-/-^, *rag2*^-/- ^*stat1*^-/-^, and *rag2*^-/- ^*ifnar*^-/- ^mice relative to wild-type mice at 72 hours p.i. (Table 2). *Stat1*^-/- ^mice, *rag2*^-/- ^*stat1*^-/- ^mice, and *rag2*^-/- ^*ifnar*^-/- ^mice died of a typical viral encephalitis 8 to 9 days p.i., based on the symptoms of hunched posture, ataxia, and hyperexcitability, which preceded death by ~18 hours (Table 2). *Ifnar*^-/- ^*ifngr*^-/- ^mice succumbed to KOS-GFP infection just 5.2 ± 0.1 days p.i. (Table 2), and presented with acute lethargy ~8 hours prior to death. Consistent with the findings of Luker, et al. [19], dissection at the time of death revealed that the livers of *ifnar*^-/- ^*ifngr*^-/- ^mice were visibly discolored and the entire liver mass was GFP^+ ^(not shown). Thus, fulminant viral infection of the liver, and presumably liver failure, appeared to be the primary cause of death in KOS-GFP-infected *ifnar*^-/- ^*ifngr*^-/- ^mice.

**Table 2:** Effect of interferon receptors versus Stat 1 on HSV-1 shedding and the survival of infected mice.

		**Viral titers per eye on day 3 p.i.^c^**	**Survival**	
Virus^a^	Mouse strain^b^		Frequency^d^	Duration (days)^e^
	wild-type	1.8 ± 0.3	100%	> 60
	*rag2*^-/-^	1.5 ± 0.4	0%	18 ± 0.5
	*ifngr*^-/-^	1.8 ± 0.5	100%	> 60
	*ifnar*^-/-^	2.8 ± 0.3	50%	11 ± 1^‡^
KOS-GFP (ICP0^+^)	*ifnar*^-/- ^*ifngr*^-/-^	3.6 ± 0.1*	0%	5.2 ± 0.1^‡^
	*stat1*^-/-^	4.3 ± 0.1*	0%	8.1 ± 0.3^‡^
	*rag2*^-/- ^*stat1*^-/-^	4.0 ± 0.2*	0%	8.6 ± 0.2^‡^
	*rag2*^-/- ^*ifnar*^-/-^	4.2 ± 0.2*	0%	7.9 ± 0.2^‡^
	
	wild-type	1.1 ± 0.6	center100%	> 60
	*rag2*^-/-^	0.7 ± 0.5	100%	> 60
	*ifngr*^-/-^	1.4 ± 0.4	100%	> 60
	*ifnar*^-/-^	2.2 ± 0.1	100%	> 60
0^-^-GFP (ICP0^-^)	*ifnar*^-/- ^*ifngr*^-/-^	3.4 ± 0.3*	16%	11 ± 1^‡^
	*stat1*^-/-^	4.4 ± 0.1*	50%	11 ± 1^‡^
	*rag2*^-/- ^*stat1*^-/-^	3.4 ± 0.2*	0%	11 ± 1^‡^
	*rag2*^-/- ^*ifnar*^-/-^	3.8 ± 0.2*	0%	10 ± 1^‡^

^a ^Mice were inoculated with 2 × 10^5 ^pfu per eye of HSV-1 strain KOS-GFP or 0^-^-GFP.

^b ^The relationship of the genotype and phenotype of these mice is defined in Table 1.

^c ^The mean ± standard error of the mean of the logarithm of infectious viral titers recovered from the ocular tear film of mice at day 3 p.i. (n = 6 per group).

^d ^The percentage of mice that survived until day 60 p.i.

^e ^The mean ± standard error of the mean duration of survival of those mice that survived for less than 60 days after inoculation with HSV-1. Groups of mice in which no deaths were recorded were sacrificed on day 60 p.i., and their duration of survival is indicated as "> 60."

* p < 0.05 that viral titers on day 3 p.i. were equivalent to those recovered from wild-type mice infected with the same virus, based on one-way ANOVA and Tukey's post hoc t-test.

^‡ ^p < 0.05 that the duration of survival was equivalent to the duration of survival of *rag2*^-/- ^(lymphocyte-deficient) mice infected with the same virus, based on one-way ANOVA and Tukey's post hoc t-test.

Following inoculation with 2 × 10^5 ^pfu per eye of 0^-^-GFP, similar levels of GFP reporter gene expression from the ICP0 gene (diagram in Fig. 4) were observed in the corneas of mice at 36 hours p.i. (Fig. 7B). By 60 and 84 hours p.i., GFP fluorescence decreased to nearly undetectable levels in the corneas of wild-type, *rag2*^-/-^, and *ifngr*^-/- ^mice (Fig. 7B). All mice with a defect in the *ifnar *or *stat1 *genes failed to limit 0^-^-GFP spread, and GFP fluorescence was still evident throughout the cornea at 84 hours p.i. (Fig. 7B). Likewise, 0^-^-GFP titers were an average 300 to 1000 times higher in the tear film of *ifnar*^-/- ^*ifngr*^-/-^, *stat1*^-/-^, *rag2*^-/- ^*stat1*^-/-^, and *rag2*^-/- ^*ifnar*^-/- ^mice relative to wild-type mice at 72 hours p.i. (Table 2). Most of the mice that shed titers of >1000 pfu per eye of 0^-^-GFP on day 3 p.i. died of the infection. *Rag2*^-/- ^mice survived 0^-^-GFP-infection for 60 days and exhibited no symptoms of disease. In contrast, *rag2*^-/- ^*stat1*^-/- ^mice and *rag2*^-/- ^*ifnar*^-/- ^mice uniformly succumbed to 0^-^-GFP infection (Table 2). Thus, the IFN-α/β receptor and downstream Stat 1 transcription factor are essential for the innate host response that represses 0^-^-GFP replication in *rag2*^-/- ^mice.

### Stat 1 is not essential for the early synthesis of HSV-1 latency-associated transcripts

A subset of neurons synthesize LAT RNAs as soon as HSV-1 infection spreads to the TG [22]. The relative frequency of LAT^+ ^neurons and viral antigen (Ag)^+ ^neurons was compared in the TG of wild-type mice, *rag2*^-/- ^mice, *stat1*^-/- ^mice, or *rag2*^-/- ^*stat1*^-/- ^mice inoculated with HSV-1 strain KOS (2 × 10^5 ^pfu per eye). On days 3 and 5.5 p.i., the frequency of LAT^+ ^neurons was equivalent in all strains of mice, and approximately 1 to 3 LAT^+ ^neurons were observed for every 1000 TG neurons counted (Table 3). Thus, the process by which HSV-1 rapidly establishes latent infections in this subset of neurons is not dependent on lymphocytes (as previously shown; Ref. 22) or the Stat 1 signaling pathway.

**Table 3:** Abundance of LAT^+ ^versus HSV antigen^+ ^neurons in trigeminal ganglia infected with HSV-1 strain KOS.

	**Day 3**		**Day 5.5**	
Mice^**a**^	LAT^+ ^neurons^**b**^	HSV Ag^+ ^neurons^**c**^	LAT^+ ^neurons	HSV Ag^+ ^neurons
		
wild-type	0.02 – 0.04% (12)^**d**^	7 – 15% (4399)	0.1 – 0.2% (45)	9 – 18% (5300)
*rag2*^-/-^	0.02 – 0.04% (12)	10 – 20% (6122)	0.1 – 0.3% (82)	18 – 36% (10897)
*stat1*^-/-^	0.03 – 0.06% (18)	10 – 20% (5978)	0.1 – 0.2% (48)	> 20% (TNTC)^**e**^
*rag2*^-/- ^*stat1*^-/-^	0.02 – 0.04% (11)	8 – 17% (5005)	0.1 – 0.2% (59)	> 20% (TNTC)

^**a**^Mice were inoculated with 2 × 10^5 ^pfu per eye of wild-type HSV-1 strain KOS, and were sacrificed 72 hours (Day 3) or 132 hours (Day 5.5) after inoculation to measure the relative abundance of trigeminal ganglion neurons that expressed HSV-1 LAT RNA versus viral antigens.

^**b**^Neurons in 7 μM sections of KOS-infected trigeminal ganglia (TG) that hybridized with a latency-associated transcript (LAT)-specific riboprobe and which were HSV antigen-negative, as determined under a fluorescent microscope.

^**c**^Neurons in 7 μM sections of KOS-infected trigeminal ganglia (TG) that were labeled by rabbit anti-HSV antibody and which were LAT-negative, as determined under a fluorescent microscope.

^**d**^The estimated percent of TG neurons that were LAT^+^, or HSV antigen^+^, is based on the total number of neurons counted (number shown in parentheses) in a total of 144 TG sections derived from 6 independent TG (i.e., 24 sections per TG were analyzed). It was estimated that this number of sections contained at least 30,000 neurons but not more than 60,000 neurons. The reported range of percentages of LAT^+ ^or HSV antigen^+ ^neurons was estimated by dividing the number of counted neurons by these lower or upper limits (30,000 or 60,000), and by multiplying times 100 to convert to a percentage.

^**e**^TNTC, too numerous to count.

During the acute infection, HSV Ag^+ ^neurons were >100-fold more abundant in TG than LAT^+ ^neurons (Table 3). On day 3 p.i., the frequency of HSV Ag^+ ^neurons was equivalent in all groups of TG, and ~100 to 300 HSV Ag^+ ^neurons were observed for every 1000 TG neurons analyzed. On day 5.5 p.i., HSV Ag^+ ^neurons were twice as abundant in the TG of *rag2*^-/- ^mice relative to strain 129 mice (Table 3). On day 5.5 p.i., HSV Ag^+ ^neurons were too numerous to count in the TG of *stat1*^-/- ^and *rag2*^-/- ^*stat1*^-/- ^mice and viral CPE severely compromised the integrity of the tissue. Thus, consistent with other results, the Stat 1 signaling pathway was essential to restrict the spread of wild-type HSV-1 from the site of inoculation to the TG.

### An ICP0^- ^virus establishes latent infections in the trigeminal ganglia of mice

The relative efficiency with which an ICP0^- ^virus establishes latent infection in the TG of wild-type mice, *ifnar*^-/- ^mice, *ifngr*^-/- ^mice, or *stat1*^-/- ^mice was compared to wild-type HSV-1 strain KOS. All strain 129 mice inoculated with 2 × 10^5 ^pfu per eye of KOS survived the acute infection, as did all strain 129 mice, *ifnar*^-/- ^mice, or *ifngr*^-/- ^mice inoculated with 2 × 10^5 ^pfu per eye of 0^-^-GFP (Table 4). Despite a ten-fold reduction in viral inoculum, 100% of *rag2*^-/- ^*ifnar*^-/- ^mice, 79% of *ifnar*^-/- ^*ifngr*^-/- ^mice, and 50% of *stat1*^-/- ^mice succumbed to acute infection following inoculation with 2 × 10^4 ^pfu per eye of 0^-^-GFP (Table 4).

**Table 4:** Efficiency with which wild-type HSV-1 and ICP0^- ^viruses reactivate from latently infected trigeminal ganglia.

		**ACUTE INFECTION**		**REACTIVATION**		
				
Mouse strain	Virus^a^	Inoculum^b^	Survival^c^	Viral spread to L7 cells^d^	Virus in TG on Day 14^e^	Viral genomes per TG^f^
Strain 129	KOS	2 × 10^5^	100% (n = 15)	11/16	14/16	3.0 × 10^5^
Strain 129	0^-^-GFP	2 × 10^5^	100% (n = 15)	0/16	0/16	1.3 × 10^5 ^*
*ifngr*^-/-^	0^-^-GFP	2 × 10^5^	100% (n = 25)	0/38	0/38	1.2 × 10^5 ^*
*ifnar*^-/-^	0^-^-GFP	2 × 10^5^	100% (n = 21)	0/16	2/16	3.5 × 10^5^
*rag2*^-/- ^*ifnar*^-/-^	0^-^-GFP	2 × 10^4^	0% (n = 11)	ND^h^	ND	ND
*ifnar*^-/- ^*ifngr*^-/-^	0^-^-GFP	2 × 10^4^	21% (n = 14)	0/6	0/6	ND
*stat1*^-/-^	0^-^-GFP	2 × 10^4^	50% (n = 50)	0/22	2/22	1.8 × 10^5^

^a ^Virus used to inoculate mice.

^b^Dose of virus in 4 μl viral inoculum applied to each eye (i.e., pfu per eye).

^c^The percentage of mice that survived until Day 38 p.i., when mice were first sacrificed for experiments.

^d^Frequency of KOS or 0^-^-GFP reactivation in latently infected trigeminal ganglia, as determined by the development of cytopathic effect in monolayers of L7 cells co-cultured with ganglion explants.

^e^Frequency of KOS or 0^-^-GFP reactivation from latently infected trigeminal ganglia, as determined by the presence of infectious virus in trigeminal ganglion homogenates on day 14 post explant.

^f^The average number of viral genomes per TG, as determined by competitive PCR analysis of TG DNA harvested from each treatment group of mice (per the results presented in Figure 8).

^h^Not determined because too few mice survived the acute infection.

* p < 0.05 that 0^-^-GFP genome loads in latently infected TG were equivalent to wild-type levels of viral genomes in KOS latently-infected TG, based on a two-way t-test.

**Figure 8:**
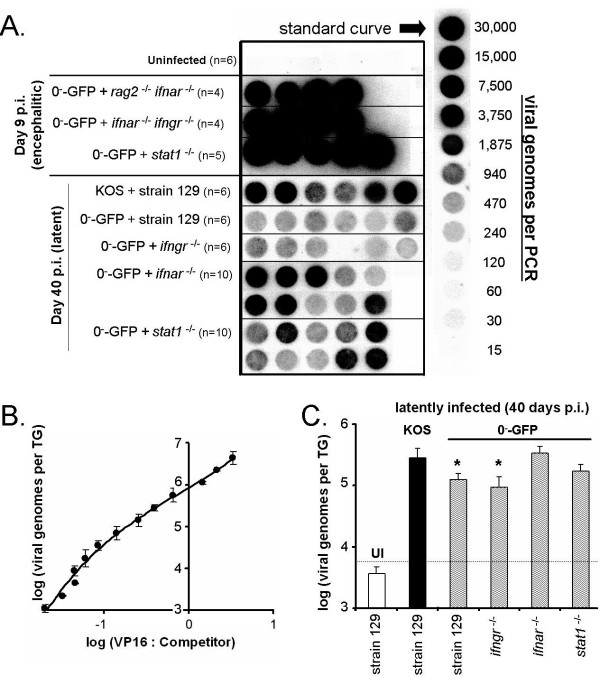
**Measurement of KOS and 0^-^-GFP viral genome loads in the trigeminal ganglia of HSV-1 latently infected mice**. **A**. Dotblot of HSV-1 VP16 PCR products. Each "dot" contains VP16 PCR product amplified from the TG DNA of a single mouse, and the n-values indicate numbers of mice per group. TG harvested from uninfected (UI) mice served as negative controls for the PCR. TG harvested from mice dying of encephalitis (Day 9 p.i.) belonged to one of the following groups: *rag2*^-/- ^*ifnar*^-/- ^mice, *ifnar*^-/- ^*ifngr*^-/- ^mice, or *stat1*^-/- ^mice inoculated with 2 × 10^4 ^pfu per eye of 0^-^-GFP. TG harvested from mice that were latently infected with HSV-1 (Day 40 p.i.) belonged to one of the following groups: strain 129 mice inoculated with 2 × 10^5 ^pfu per eye of KOS; strain 129 mice, *ifngr*^-/- ^mice, or *ifnar*^-/- ^mice inoculated with 2 × 10^5 ^pfu per eye of 0^-^-GFP; or *stat1*^-/- ^mice inoculated with 2 × 10^4 ^pfu per eye of 0^-^-GFP. The standard curve on the right consists of PCR products amplified from a two-fold dilution series of VP16 plasmid DNA. **B**. The ratio of yields of VP16 to competitor PCR product yields (competitor dotblot not shown) was used to estimate viral genome copy number per PCR. The logarithm of *viral genomes per TG*, y, was plotted as a function of the mean logarithm of *the ratio of VP16 PCR product yield: competitor PCR product yield*, x, amplified from duplicate PCRs of each dilution of VP16 plasmid (error bars indicate the standard deviation between duplicate PCRs). The relationship between viral genome load and PCR product yields was described by the equation, y = 0.2556•x^3 ^+ 0.1055•x^2 ^+ 1.2079•x + 5.9309 (r^2 ^= 0.99). The number of HSV-1 genomes per TG in each sample was derived from fitting the data shown in panel A to the standard curve shown in panel B. **C**. Number of HSV-1 genomes per TG in mice that were uninfected or were latently infected with KOS or 0^-^-GFP. The dashed line indicates the lower limit of detection of the PCR assay. Asterisks denote significant differences in viral genome load per TG relative to strain 129 mice latently infected with KOS (p < 0.05, two-way t-test).

HSV-1 genome loads per TG were analyzed by competitive PCR amplification of a virion protein 16 (VP16) gene sequence. VP16 PCR products were not amplified from uninfected TG DNA, but were consistently amplified from HSV-1 infected TG DNA samples (Fig. 8A). VP16 PCR products amplified from a VP16 plasmid DNA dilution series defined the relationship between PCR product yield and viral genome copy number per PCR (Fig. 8A and 8B). Viral genome load per TG was evaluated in 0^-^-GFP infected mice that developed encephalitis at day 9 p.i. (Fig. 8A). VP16 PCR product amplification was well outside the quantitative range of the PCR assay, but demonstrated that the TG of encephalitic *rag2*^-/- ^*ifnar*^-/- ^mice, *ifnar*^-/- ^*ifngr*^-/- ^mice, or *stat1*^-/- ^mice all possessed in excess of 10^7 ^HSV-1 genomes per TG on day 9 p.i. (Fig. 8A).

HSV-1 latently infected mice were sacrificed at day 40 p.i. to measure viral genome load per TG. KOS-latently infected strain 129 mice contained an average of 3.0 × 10^5 ^viral genomes per TG (Fig. 8C; Table 4). In strain 129 mice and *ifngr*^-/- ^mice, the number of latent 0^-^-GFP genomes per TG was ~40% of the wild-type level achieved by KOS. In *ifnar*^-/- ^mice, the number of latent 0^-^-GFP genomes per TG was ~115% of the wild-type level. In *stat1*^-/- ^mice (inoculated with a ten-fold lower dose of 0^-^-GFP), the number of latent 0^-^-GFP genomes per TG was ~60% of the wild-type level (Fig. 8C; Table 4). If one considers the primary data, 0^-^-GFP established latent infections in 50% of *ifnar*^-/- ^mice and *stat1*^-/- ^mice that met or exceeded the latent viral genome load per TG achieved by KOS in strain 129 mice (Fig. 8A).

### An ICP0^- ^virus reactivates inefficiently from latently infected trigeminal ganglia

The efficiency with which latent KOS (ICP0^+^) and 0^-^-GFP (ICP0^-^) reactivated from TG explants was compared. TG were harvested from mice on days 38 and 39 p.i., heat stressed at 43°C for 3 hours, co-cultured with L7 cell monolayers for 14 days, and were homogenized on day 14 to test for the presence of infectious virus within each TG. Of the KOS-latently infected TG harvested from strain 129 mice, 11 of 16 TG explants produced infectious virus that spread to co-cultured L7 cells (Table 4), and 14 of 16 TG homogenates contained infectious KOS on day 14 post explant (Table 4). Of the 0^-^-GFP-latently infected TG harvested from strain 129 mice, *ifngr*^-/- ^mice, *ifnar*^-/- ^mice, *ifnar*^-/- ^*ifngr*^-/- ^mice, or *stat1*^-/- ^mice, 0 of 98 TG explants produced infectious virus that spread to co-cultured L7 cells (Table 4). In contrast, infectious 0^-^-GFP was detected in 2 of 16 *ifnar*^-/- ^TG homogenates and 2 of 22 *stat1*^-/- ^TG homogenates on day 14 post explant (Table 4). Reactivation of 0^-^-GFP was detected in none of the other TG homogenates on day 14 post explant (Table 4). Thus, consistent with previous results, even when wild-type HSV-1 and ICP0^- ^viruses establish equivalent latent infections (e.g., *ifnar*^-/- ^mice; Table 4), ICP0^- ^viruses are severely impaired in their capacity to reactivate from latently infected TG explants [23,24].

### Prior infection with an avirulent ICP0^- ^virus induces protective immunity against HSV-1

**Figure 9:**
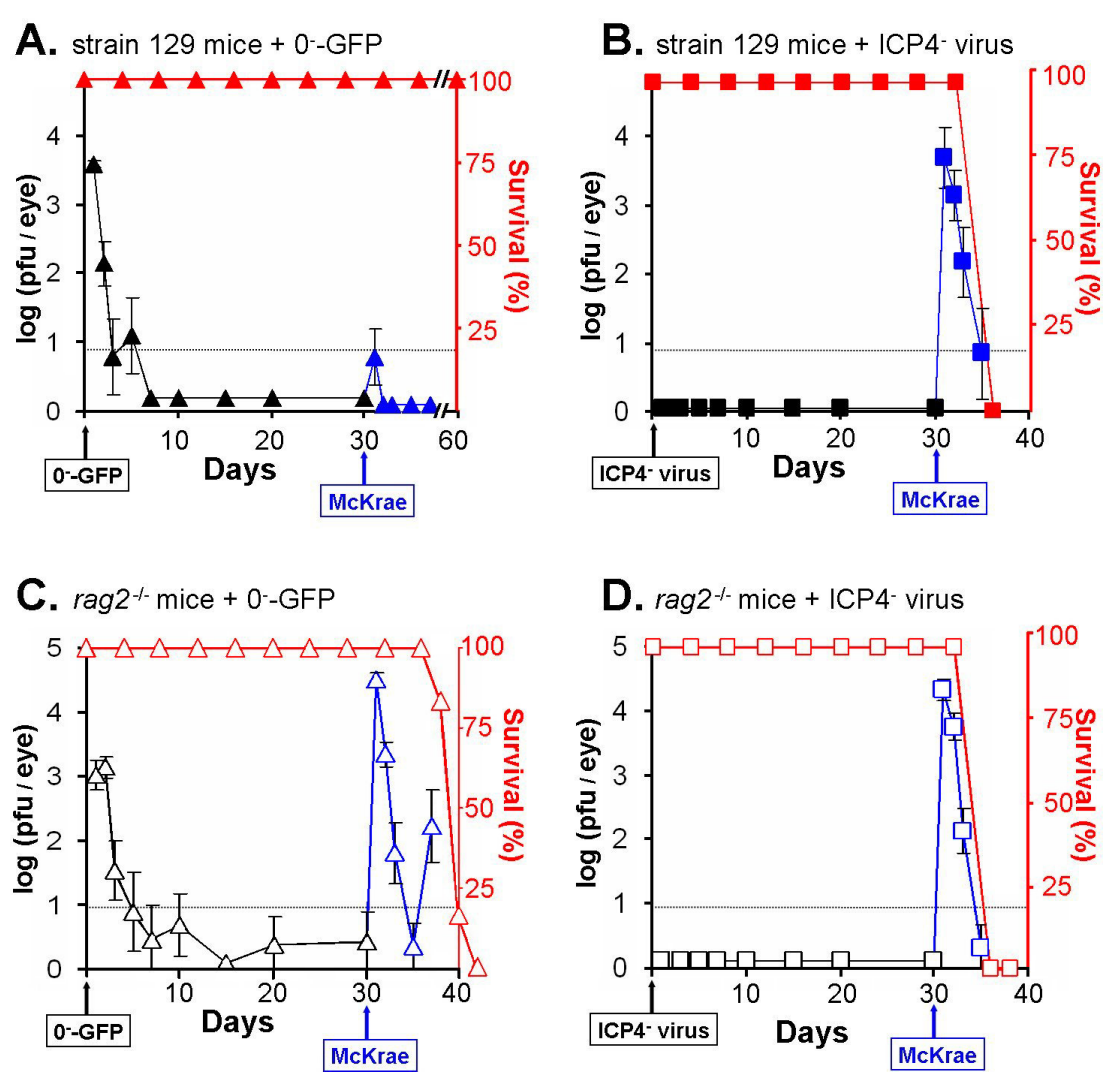
**An ICP0^- ^virus induces a protective immune response against HSV-1**. Strain 129 mice were inoculated with 2 × 10^5 ^pfu per eye of the **A**. ICP0^- ^virus 0^-^-GFP or **B**. the ICP4^- ^virus n12 (n = 6 mice per group). *Rag2*^-/- ^mice were inoculated with 2 × 10^5 ^pfu per eye of the **C**. ICP0^- ^virus 0^-^-GFP or **D**. the ICP4^- ^virus n12 (n = 6 mice per group). The mean ± sem of the logarithm of viral titers recovered from mouse eyes is plotted over time (black symbols). The survival of 0^-^-GFP-infected mice and ICP4^- ^virus-infected mice is plotted over time (red symbols). On day 30 p.i., 0^-^-GFP-infected and ICP4^- ^virus-infected mice were secondarily challenged with 2 × 10^5 ^pfu per eye of HSV-1 strain McKrae. Viral titers recovered from the eyes of strain 129 mice or *rag2*^-/- ^mice after secondary challenge with McKrae is plotted over time (blue symbols). Dashed lines indicate the lower limit of detection of the plaque assay.

Interferon-sensitive ICP0^- ^viruses have not been considered for their potential to vaccinate against herpetic disease. The efficacy with which an ICP0^- ^virus or ICP4^- ^virus induced protective immunity against lethal HSV-1 challenge was compared. Strain 129 mice were inoculated with 2 × 10^5 ^pfu per eye of 0^-^-GFP or an ICP4^- ^virus. The ICP0^- ^virus replicated transiently, but titers of 0^-^-GFP in ocular tear film remained low to undetectable between days 3 and 30 p.i. (Fig. 9A), and the ICP4^- ^virus failed to replicate (Fig. 9B). As anticipated, 100% of 0^-^-GFP- or ICP4^- ^virus-infected mice survived and remained healthy for 30 days p.i. (Fig. 9A, 9B). On day 30 p.i., these mice were challenged with 2 × 10^5 ^pfu per eye of HSV-1 strain McKrae. In 0^-^-GFP-infected strain 129 mice, one of six mice shed detectable virus at 24 hours post-challenge (Fig. 9A). All of the 0^-^-GFP-infected mice remained healthy until the conclusion of the experiment, 30 days after secondary challenge with McKrae (Fig. 9A). In ICP4^- ^virus-infected mice, McKrae replicated to high titers in the eyes of mice, and progressed to fatal encephalitis within 5.8 ± 0.2 days (Fig. 9B).

Equal numbers of *rag2*^-/- ^mice were included in this experiment. Inoculation of *rag2*^-/- ^mice with 0^-^-GFP or the ICP4^- ^virus produced an equivalent pattern of viral shedding as was observed in strain 129 mice between days 1 and 30 p.i. (Fig. 9C and 9D). On day 30 p.i., *rag2*^-/- ^mice were secondarily challenged with McKrae. McKrae replicated to high titers in *rag2*^-/- ^mice that were first inoculated with 0^-^-GFP or the ICP4^- ^virus (Fig. 9C and 9D). In ICP4^- ^virus-infected *rag2*^-/- ^mice, McKrae infection progressed to fatal encephalitis by 5.7 ± 0.1 days post challenge (Fig. 9D). In 0^-^-GFP-infected *rag2*^-/- ^mice, McKrae infection required 9.0 ± 0.5 days to progress to fatal disease (Fig. 9C), suggesting that the ongoing innate response to 0^-^-GFP infection delayed the progression of McKrae infection to a fatal encephalitis. Therefore, while 0^-^-GFP infection is avirulent in *rag2*^-/- ^mice, 0^-^-GFP cannot induce protective immunity against HSV-1 in animals that lack a lymphocyte-driven adaptive immune response.

## Discussion

### IFN receptors and Stat 1 are integral to innate host control of HSV-1 infection

Loss of IFN-α/β receptors in *ifnar*^-/- ^mice compromised innate resistance to HSV-1, but the defect was not as profound as observed in *stat1*^-/- ^mice. Thus, on day 3 p.i., *ifnar*^-/- ^mice shed 30- to 100-times less infectious virus per eye than *stat1*^-/- ^mice (Table 2). Likewise, *ifnar*^-/- ^mice uniformly survived ICP0^- ^viral infections with little disease (Table 2), whereas 50% of *stat1*^-/- ^mice died of 0^-^-GFP infection and all of the survivors were frankly diseased. The capacity of *rag2*^-/- ^mice to repress 0^-^-GFP replication was clearly dependent on the IFN-α/β receptor and Stat 1, because 0^-^-GFP produced uniformly fatal infections in *rag2*^-/- ^*ifnar*^-/- ^mice and *rag2*^-/- ^*stat1*^-/- ^mice. Mice that lacked IFN-α/β receptors ***and ***IFN-γ receptors failed to suppress the replication of wild-type or ICP0^- ^viruses at the site of inoculation. Because the extent of the defect was similar to that observed in *stat1*^-/- ^mice, the combined activities of IFN-α/β and IFN-γ appear to activate the Stat 1-dependent host response that limits HSV-1 spread *in vivo*.

The innate resistance of host structural cells to HSV-1 appears to be compromised in the absence of Stat 1 or IFN-α/β receptors, presumably because these cells fail to mount an antiviral state (Fig. 7). However, this simple explanation does not account for all of the observations presented herein. For example, HSV-1 produced a rapid and fatal disease in *ifnar*^-/- ^*ifngr*^-/- ^mice, which as described by Luker, et al. [19], appeared to be a viral hepatitis. HSV-1 infection rarely causes hepatitis in humans [25], and liver infection is not a prominent feature in animal models. Moreover, *stat1*^-/- ^mice infected with wild-type HSV-1 died of encephalitis two to three days later. This divergence in the outcomes of infection in *ifnar*^-/- ^*ifngr*^-/- ^mice and *stat1*^-/- ^mice draws attention to an important caveat of studies in knockout mice: despite the targeted nature of the genetic mutations, loss of a single protein may have pleiotropic effects. For example, basic immune functions such as hematopoiesis or leukocyte activation may be altered by combinations of these mutations. Alternatively, the non-permissiveness of leukocytes for HSV-1 replication may be dependent on the combined activities of IFN-α/β receptors and IFN-γ receptors [26,27]. Thus, the fulminant liver infection that develops in HSV-1-infected *ifnar*^-/- ^*ifngr*^-/- ^mice may be due to uncontrolled HSV-1 replication in at least one leukocyte subset, which would confer upon HSV-1 the unusual property of blood-borne spread to the liver. Given such possibilities, the results of the current study must be interpreted with a certain degree of caution.

### Stat 1 is not essential for the early synthesis of HSV-1 latency-associated transcripts

A subset of neurons express LAT RNAs, but not viral proteins, when HSV-1 first enters the TG on day 3 after ocular inoculation [22]. Comparison of the numbers of LAT^+ ^neurons in *stat1*^+/+ ^versus *stat1*^-/- ^mice indicated that this phenomenon is not Stat 1-dependent (Table 3). Thus, the Checkpoint Model fails to explain how HSV-1 establishes latent infections in the subpopulation of *stat1*^-/- ^neurons that are LAT^+ ^on days 3 and 5.5 p.i. (Table 3). Although LAT^+ ^neurons were certainly present in *stat1*^-/- ^mice, HSV Ag^+ ^neurons were 100- to 500-fold more abundant between days 3 and 5.5 p.i. (Table 3). On day 5.5 p.i., *stat1*^-/- ^and *rag2*^-/- ^*stat1*^-/- ^mice were diseased, their TG were beginning to hemorrhage, and the animals were within 24 hours of death. Clinically, herpesviral latency refers to the absence of infectious virus and disease in a host, and requires the repression of viral replication in the *entire population *of virus-infected cells in the body. Thus, although a small subset of neurons represses HSV-1 replication in a Stat 1-independent manner, we conclude that both Stat 1 and lymphocytes are required for a latent HSV-1 infection to be established at the clinically relevant level of the host organism.

### ICP0 antagonizes Stat 1-dependent repression of herpes simplex virus

ICP0^- ^viruses are rapidly repressed in *scid *or *rag2*^-/- ^mice. The failure of ICP0^- ^viruses to sustain replication in lymphocyte-deficient mice is not due to a leaky adaptive immune response, nor is this a non-specific phenotype of any HSV-1 mutant. For example, VP16^- ^and thymidine kinase^- ^mutants of HSV-1 establish slowly progressing infections that are fatal in *scid *mice [28,29]. Rather, it appears that an innate host response that is dependent upon the IFN-α/β receptor and Stat 1 potently represses the replication of ICP0^- ^viruses in *scid *or *rag2*^-/- ^mice.

It is generally believed that ICP0 functions as an activator in the balance between HSV-1 latency and reactivation, and that ICP0 acts to prevent host cells from repressing HSV-1 replication *in vitro *[5,6,30]. However, a credible explanation has been lacking to explain how synthesis of ICP0, or lack thereof, can globally effect whether or not HSV-1 initiates productive viral replication. The results of the current study suggest a potentially simple explanation: *when ICP0 is synthesized*, the protein overcomes Stat 1-dependent cellular repression of HSV-1 such that the virus can complete its replication cycle; *when ICP0 is not synthesized*, Stat 1-dependent cellular repression silences the HSV-1 genome.

Given ICP0's capacity to function as a viral IFN antagonist *in vitro *[9,10,14,15], we infer that ICP0 most likely functions in virus-infected cells to prevent an IFN-inducible block to viral replication (Checkpoint 1, Fig. 1C). Although PML has been proposed to serve a critical role in repressing ICP0^- ^viruses [21], our findings in *PML*^-/- ^mice do not support a direct role for PML in the process by which host cells repress ICP0^- ^viruses *in vivo*. Therefore, it remains to be determined how ICP0 antagonizes Stat 1-dependent repression of HSV-1.

### Implications for the regulation of viral latency

The available evidence suggests that the 9.3 kb R_L _region, which contains the ICP0 gene, plays a pivotal role in regulating HSV-1 latency (Fig. 4). The evidence that supports this point is summarized: ***1***. Synthesis of ICP0 promotes HSV-1 reactivation in latently infected TG neurons [24,31]; ***2***. The antisense LAT gene is the only viral gene transcribed during latency and produces stable 1.5 and 2.0 kb introns that accumulate in neurons [32,33]; ***3***. Synthesis of ICP34.5 is necessary for HSV-1 to complete its replication cycle in neurons [34-36]; and ***4***. De-repression of the antisense L/ST gene produces an HSV-1 mutant that overexpresses L/ST RNA and is replication-impaired in neurons despite an intact ICP34.5 open-reading frame [3].

Failure to synthesize the R_L_-encoded viral IFN antagonists, ICP0 or ICP34.5, is a possible mechanism by which HSV-1 may cease productive replication *in vivo *(Fig. 1C). Failure to synthesize ICP0 renders HSV-1 vulnerable to repression by the host IFN response. Likewise, failure to synthesize ICP34.5 renders HSV-1 vulnerable to innate immune repression in *scid *mice [37], and this repression is dependent on IFN-α/β receptors and PKR [11]. If synthesis of ICP0 and ICP34.5 is necessary to promote HSV-1 replication *in vivo*, then it is possible that the LAT and L/ST genes provide HSV-1 with an efficient mechanism to pro-actively halt the synthesis of ICP0 or ICP34.5, such that HSV-1 can rapidly cease productive replication *in vivo*.

This hypothesis may seem counterintuitive. Why would HSV-1 carry two genes that promote the IFN-induced repression of the viral genome? Therefore, it is worth noting that herpesviruses are presented with two choices at the height of an immune response: **1**. cytolytic destruction of the host cell by CD8^+ ^T cells, or **2**. cessation of viral antigen synthesis before CD8^+ ^T cells can destroy the host cell. Given the arrangement of genes in the R_L _region (Fig. 4), it is possible that the LAT and/or L/ST genes may serve to ensure the timely shutoff of HSV-1 replication and antigen synthesis when the local immune response exceeds a critical threshold in an HSV-1 infected tissue. This hypothesis may be relevant in explaining: ***i***. how viral antigen synthesis decreases from high to undetectable levels within 8 hours after T cells infiltrate HSV-1 infected ganglia [38]; ***ii***. how HSV-1 infected neurons avoid cytolytic destruction by T cells [39,40]; and ***iii***. why neurons infected with LAT^- ^viruses undergo apoptosis (cytolysis?) more frequently than neurons infected with LAT^+ ^viruses [41-43]. Given the dearth of evidence that the LAT or L/ST genes actually antagonize the synthesis of ICP0 and ICP34.5, further work will be required to address these hypotheses.

### Clinical implications

HSV-1 becomes hypersensitive to IFN-inducible repression in the absence of ICP0 or ICP34.5 [16]. Given the extensive similarity between HSV-1 and HSV-2, the results suggest a logical approach to develop a live, attenuated vaccine against genital herpes. When introduced at the peripheral epithelium, ICP0^- ^viruses are incapable of producing disease in lymphocyte-deficient *scid *or *rag2*^-/- ^mice. Despite their avirulence, ICP0^- ^viruses establish latent infections in the ganglia that innervate the site of inoculation (Fig. 8) and elicit an adaptive immune response that protects immunocompetent mice against virulent HSV-1 (Fig. 9). In contrast, a replication-defective ICP4^- ^virus fails to elicit any semblance of protective immunity.

The advantage of a live, attenuated HSV vaccine would be two-fold: **1**. the vaccine strain would be antigenically identical to wild-type HSV with the exception of a single mutated open-reading frame (e.g., ICP0), and **2**. the latent infection established by such a virus might boost the adaptive immune response over time through periodic synthesis of viral antigen at the single cell level [44,45]. Failure to sustain a broad-based immune response over time likely accounts for the failure of subunit vaccines or replication-defective viruses to protect against genital herpes [46,47]. While a live HSV vaccine is often considered unsafe because the vaccine strain would establish a latent infection in the recipient, such claims ignore the natural history of HSV infections [48,49]. About 4 billion people currently harbor latent HSV-1 and/or HSV-2 in their nervous system, and ~3 billion of these infections are asymptomatic. Further investigation will be required to determine if ICP0^- ^strains of HSV-2 can be used to achieve an appropriate balance between safety and antigenicity in developing a live, attenuated vaccine against genital herpes.

## Conclusion

In recent years, it has become increasingly evident that the host immune response plays a more direct role in the maintenance of HSV-1 latency than previously envisioned [50,51]. The results of the current study add to this body of evidence, and suggest that the antagonistic actions of host IFNs and the viral IFN antagonist ICP0 may help HSV-1 "choose" between one of its two programs of gene expression, productive replication or latent infection. The proposed Checkpoint Model (Fig. 1C) represents an initial attempt to explain these observations, and will require extensive testing to determine its validity.

Despite its limitations, the Checkpoint Model offers a useful conceptual framework from which to begin considering whether these observations are really unique to HSV-1. Most persistent viruses possess the capacity to alternate between rapid, slow, and/or non-existent modes of productive replication. Therefore, it is worth considering that perhaps other persistent viruses also encode "activators" such as ICP0 whose synthesis (or lack thereof) regulates the rate of productive replication *in vivo *by virtue of antagonizing a host repressor that has been present since the dawn of life: the innate host response to viral infections.

## Methods

### Cells and viruses

Vero cells and L7 cells [52] were propagated in Dulbecco's Modified Eagle's medium (DMEM) containing 0.15% HCO_3_^- ^supplemented with 5% fetal bovine serum (FBS), penicillin G (100 U/ml), and streptomycin (100 mg/ml), hereafter referred to as "complete DMEM." Wild-type HSV-1 strains KOS, KOS-GFP, and McKrae were propagated in Vero cells cultured in complete DMEM. The ICP0^- ^virus n212 [53,54] contains a 14 bp insertion (CTAGACTAGTCTAG) in codon 212 of the ICP0 gene of HSV-1 strain KOS, which inserts stop codons into all three open-reading frames of the ICP0-encoding DNA strand (Fig. 4). The n212 (ICP0^-^) virus was propagated and titered in ICP0-complementing L7 cells [52]. The ICP4^- ^virus n12 contains a 16 bp insertion (GGCTAGTTAACTAGCC) in codon 262 of the ICP4 gene of HSV-1 strain KOS, which inserts stop codons into all three open-reading frames of the ICP4-encoding DNA strand. The n12 (ICP4^-^) virus was propagated and titered in E5 cells [55] (generously provided by Priscilla Schaffer, Harvard University).

KOS-GFP is a recombinant virus derived from HSV-1 strain KOS that expresses green fluorescent protein (GFP) from a CMV promoter-GFP gene cassette inserted in the intergenic region at the 3' ends of the UL26 and UL27 genes, which converge from opposite strands of DNA [56]. To avoid disrupting the 3' untranslated region of either the UL26 or UL27 gene, the intergenic region (Kpn I – Fsp I; 52,733 – 53,150) was duplicated and the CMV-GFP cassette is inserted between these duplicated sequences. No known open-reading frames are disrupted by this 2.0 kbp insertion into the HSV-1 genome.

The ICP0^- ^virus 0^-^-GFP was constructed from a chimeric ICP0-GFP gene [57], in which the GFP open-reading frame was inserted into a Xho I restriction site in codon 105 of the ICP0 gene (Fig. 4). This mutation was transferred into HSV-1 strain KOS by homologous recombination. Southern blot analysis demonstrated that 0^-^-GFP possessed the desired mutation in both copies of the R_L _region (Fig. 4). Northern blot analysis confirmed that 0^-^-GFP expresses an ICP0 mRNA that migrates at ~3.1 kb as opposed to the wild-type 2.4 kb ICP0 mRNA transcribed from KOS. 0^-^-GFP is phenotypically identical to the well-established ICP0^- ^null virus n212 [53,54], based on the following observations: **1**. Both n212 and 0^-^-GFP are repressed with identical kinetics in *rag2*^-/- ^mice; **2**. Both n212 and 0^-^-GFP are virulent in *stat1*^-/- ^mice; **3**. Both n212 and 0^-^-GFP are hypersensitive to IFN-α/β *in vitro *[14]; **4**. Only 1% of n212 and 0^-^-GFP virions form plaques on Vero cells [54,58]; **5**. ICP0, but not ICP4 or VP16, provided *in trans *from adenovirus vectors allow n212 and 0^-^-GFP to efficiently form plaques on Vero cells [31], and the efficiency of plaque formation is the same as observed on ICP0-complementing L7 cells [14,31,52].

### HSV-1 infection of mice

Female mice of the strains specified in Table 1 were inoculated with HSV-1 at 6- to 10-weeks of age and were handled in accordance with the *NIH Guide for the Care and Use of Laboratory Animals*. BALB/c mice, BALB/c *scid *mice, and IFN-γ receptor null (*ifngr*^-/-^) mice were obtained from the Jackson Laboratory (Bar Harbor, ME). Strain 129 mice, *rag2*^-/- ^mice, *stat1*^-/- ^mice, and *rag2*^-/- ^*stat1*^-/- ^mice were obtained from Taconic Farms (Germantown, NY). PML^-/- ^mice were obtained from the National Cancer Institute (Frederick, MD). IFN-α/β receptor-knockout (*ifnar*^-/-^) mice and *ifnar*^-/- ^*ifngr*^-/- ^mice were obtained from B & K Universal Ltd. (East Yorkshire, United Kingdom). *Rag2*^-/- ^*ifnar*^-/- ^mice were generously provided by Nicole Meissner and Allen Harmsen (Montana State University, Bozeman). Prior to viral inoculation, mice were anesthetized by i.p. administration of xylazine (7 mg/kg) and ketamine (100 mg/kg). Mice were inoculated by scarifying the cornea with a 26-gauge needle and by placing 4 μl complete DMEM containing 2 × 10^5 ^pfu of virus on each eye.

Viral titers in the ocular tear film of mice were determined at times after inoculation by swabbing the ocular surface of both eyes with a cotton-tipped applicator, and transferring the tip into 0.4 ml complete DMEM. Viral titers were determined by a 96-well plate plaque assay on the appropriate cell line cultured in complete DMEM containing 0.5% methlycellulose (i.e., Vero cells for wild-type HSV-1, L7 cells for ICP0^- ^mutants, and E5 cells for ICP4^- ^mutants).

Titers of infectious virus in homogenates of whole eyes, trigeminal ganglia (TG), or hindbrain were determined by homogenizing tissues in 0.5 ml complete DMEM with a Pro 200 homogenizer (Pro Scientific, Oxford, CT), removing cell debris via centrifugation, and titering 10-fold serial dilutions of clarified supernatant on 24-well plates containing Vero or L7 cells. For all plaque assays, virus-infected cells were cultured in complete DMEM containing 0.5% methlycellulose for two to three days before staining with 20% methanol and 0.5% crystal violet.

GFP fluorescence in eyes, TG, and brains of mice infected with KOS-GFP was visualized on a Nikon TE2000 inverted fluorescent microscope (Nikon Instruments, Lewisville, TX) using a Jenoptik ProgRes C^10+ ^Digital Camera (JenOptik Laser, Jena, Germany). Images were collected at 2× or 4× magnification using identical exposure conditions within a given comparison group, and composite images of the brain were created by stitching together photographs that covered the ventral surface of the brain using the graphics editor, Paint Shop Pro (Jasc Software, Eden Prairie, MN). GFP fluorescence in the eyes of living mice was obtained by placing anaesthetized mice on a petri dish on the stage of the microscope.

### Analysis of HSV-1 replication in vitro

Cultures of Vero cells were established in 12-well plates at a density of 2 × 10^5 ^cells per well and were cultured in complete DMEM. Cells were treated 24 hours later by replacing the culture medium with complete DMEM containing no IFN or 200 U/ml IFN-β (PBL Biomedical Laboratories, Piscataway, NJ). Sixteen hours later, Vero cells were inoculated with 2.5 pfu per cell of wild-type HSV-1 strain KOS, 0^-^-GFP, or the ICP4^- ^virus n12. After allowing 45 minutes for adsorption, the inoculum was replaced with complete DMEM containing no IFN or 200 U/ml IFN-β. Cultures were harvested 4.5, 9.0, 13.5, or 18.0 hours after inoculation by transfer to a -80°C freezer. Upon thawing, viral titers were determined by plaque assay on appropriate indicator cells.

### Dual labeling of trigeminal ganglion sections for LAT RNA and viral proteins

Mice were euthanized and perfused with 0.1 M phosphate-buffered saline (PBS) followed by 4% paraformaldehyde. The six TG from each group of mice were immersed in 4% paraformaldehyde for 1 hour, equilibrated with 30% sucrose, embedded in Tissue-Tek^® ^O.C.T. compound (Sakura Finetechnical, Tokyo, Japan), and frozen in liquid nitrogen. Each block of tissue containing the six TG from one group of mice was cut into ~160 sections of 7 μM thickness. Slides were processed to label **1**. LAT^+ ^neurons with rhodamine, and **2**. HSV antigen^+ ^neurons with fluorescenin, as follows. LAT riboprobes, specific for bases 119629–119975 of HSV-1, were prepared at 37°C with digoxigenin RNA Labeling Mix (Roche, Mannheim, Germany). LAT riboprobe synthesis reactions were treated with DNase I and filtered through a Sephadex G-50 column prior to use. Tissue sections were incubated in a pre-hybridization buffer, and then hybridized to LAT-specific riboprobes by an overnight 45°C incubation. Tissue was washed with 2× SSC, treated with 20 μg/ml of RNase A in 2× SSC at 37°C, followed by serial washes in 0.5× SSC and 0.1× SSC. Tissue sections were equilibrated with 0.1 M PBS, and were sequentially incubated with **1**. rhodamine-labeled anti-digoxigenin Fab fragments, **2**. 3% normal rabbit serum, and **3**. fluorescein-labeled rabbit polyclonal anti-HSV antibodies (DAKO Cytomation, Carpinteria, CA) diluted 1:100 in 1% normal rabbit serum. Fluorescent-labeled tissue sections were then washed with 0.1 M PBS, and mounted under cover slips.

### Analysis of HSV-1 reactivation in trigeminal ganglion explants

Latently infected mice were sacrificed on days 38 and 39 p.i., TG were aseptically removed, and each TG was placed in one well of a 24-well plate containing 1 ml of complete DMEM. Once TG were harvested for an entire 24-well plate, the TG were heat stressed by transfer to a 43°C, 5% CO_2 _incubator for 3 hours. After heat stress, explants were transferred to a 37°C, 5% CO_2 _incubator. Twenty-four hours later, TG and cell culture medium were transferred to 24-well plates seeded 6 h earlier with 5 × 10^4 ^L7 cells per well in a volume of 0.5 ml of complete DMEM. On days 6 and 10 after explantation, TG were transferred to a 24-well plate containing freshly seeded L7 cells. After 14 days in culture, TG explants were homogenized in 500 μl of complete DMEM with a Pro 200 homogenizer (Pro Scientific) and TG homogenates were transferred to a -80°C freezer. Freeze-thawed TG homogenates were centrifuged to remove tissue debris, and 200 μl of clarified supernatant was used to inoculated each well of a 12-well plate of L7 cells (1 × 10^5 ^cells per well). After allowing 45 minutes for viral adsorption, the viral inoculum was aspirated, the cell monolayer was rinsed with 1 ml complete DMEM, and the rinse solution was replaced with 1 ml complete DMEM. Monolayers of L7 cells treated with TG homogenates were observed for six days for the development of viral cytopathic effects.

### Measurement of HSV-1 DNA load in latently infected mouse trigeminal ganglion

The left and right TG from each mouse were placed into a single 1.5 ml microfuge tube, and transferred to -80°C until the time of DNA extraction. DNA was isolated by a standard phenol: chloroform extraction procedure [59], and the number of HSV-1 genomes per TG was determined by a competitive PCR assay, which is described as follows.

The oligonucleotide primers used in the competitive PCR assay, VP16-a (5'-GGACTCGTATTCCAGCTTCAC-3') and VP16-b (5'-CGTCCTCGCCGTCTAAGTG-3'), amplified a 260-bp fragment of the HSV-1 VP16 gene. To provide an internal control for each PCR assay, a VP16 competitor template was generated by the method of Siebert and Larrick [60]. In brief, an irrelevant sequence from pUC18 was amplified with the primers VP16 mimic-a (5'-GGACTCGTATTCCAGCTTCACGGAGGACCGAAGGAG-3') and VP16 Mimic-b (5'-CGTCCTCGCCGTCTAAGTGCCAGTGCTGCAATGA), which amplify a 361-bp PCR product whose 5' ends are identical in sequence to the VP16-a and VP16-b primers. The VP16 competitor was cloned into the pCR II vector (Invitrogen Corp., Carlsbad, CA), and the resulting plasmid was used as a competitor template in the competitive PCR assay. DNA for the standard curve was obtained by diluting the plasmid pCRII: VP16 into a solution of TE buffer containing 33 ng/μl of salmon sperm DNA as carrier DNA. The most concentrated standard contained 45 fg of plasmid DNA per μl (10,000 copies per μl), and 13-serial twofold dilutions were made using TE buffer and salmon sperm DNA as the diluent.

PCR assays were conducted as follows: A solution containing 1× *Taq *buffer, 50 μM of each dNTP, 0.25 μM of each VP16 primer, 5% glycerol, and ~1500 copies of VP16 competitor template per 50 μl reaction was prepared. Forty-two microliters of this master mix were placed in 0.65 ml tubes and overlaid with mineral oil, and 100 ng of TG DNA (3 μl), DNA standards, or negative control DNA sample was added to each tube. The tubes were heated to 90°C in a thermal cycler, and 2.5 U of *Taq *polymerase diluted in 5 μl of Taq buffer was added to each sample. PCR samples were incubated for 35 cycles of 94°C for 1 minute 15 seconds, 59.5°C for 1 minute 15 seconds, and 72°C for 40 seconds.

Measurement of VP16 gene and competitor PCR product yields was performed by a modification of the dot blot procedure of Hill et al. [61]. From each amplified PCR sample, 15 μl was diluted in 500 μl of a 0.4 M NaOH solution, transferred to a 0.6 ml microfuge tube, heated to 90°C for 10 minutes, snap-cooled on ice, and blotted on Zeta Probe GT nylon membrane (BioRad Laboratories, Hercules, CA) in an 8 × 12 dotblot pattern using a Convertible™ vacuum filtration manifold (Whatman-Biometra, Gröningen, Germany). Two identical dotblots of each set of PCR samples were produced and crosslinked with 0.2 J/cm^2 ^in a UV crosslinker (Spectronics Corporation, Westbury, NY). One dotblot was hybridized to a radiolabeled oligonucleotide specific for VP16 (5'-GTCGTCGTCCGGGAGATCGAGCAGGCCCTC-3'), and the second duplicate dotblot was hybridized to a radiolabeled oligonucleotide specific for the competitor PCR product (5'-CGCTCGTCGTTTGGTATGGCTTCATTCAGC-3'). Both oligonucleotide probes were end-labeled with [α-^32^P] dATP using terminal deoxynucleotidyl transferase (Promega Corporation, Madison, WI). Each probe was allowed 16 h to hybridize to a membrane at 42°C in a solution containing 20 ng/ml labeled probe, 7% SDS, 120 mM NaH_2_PO_4_, and 250 mM NaCl. Excess probe was removed from membranes by sequential rinses in 0.1× standard saline citrate (SSC) containing 0.1% SDS and membranes were exposed to phosphor screens, which were scanned on a Cyclone PhosphorImager (Perkin Elmer Life Sciences, Boston, MA). The amount of radolabeleled probe hybridized to each dotblot was determined using OptiQuant v4.0 software (Perkin Elmer Life Sciences).

A two-fold dilution series of VP16 plasmid DNA defined the relationship between the copy number of VP16 genes in each PCR (x) and the logarithm of the ratio of VP16 to competitor PCR product yields (y) amplified from each DNA sample. The standard curve was described by the equation x = arctanh (y), but for convenience Microsoft Excel's trendline-fitting feature was used to rapidly define a third order polynomial equation (x = ay^3 ^+ by^2 ^+ cy + d) that approximates the sigmoid shape described by this equation (Fig. 8B). To estimate the copy number of viral genomes per TG, the number of HSV-1 genomes per PCR was multiplied times 150. This derivation is based on the fact that an average of 15 μg of DNA is extracted from each TG pair using an extraction procedure that recovers 50% of the total DNA. Thus, ~15 μg of DNA was present in a single TG, and the input of 0.1 μg of TG DNA per PCR contains ~1/150^th ^(0.1 μg out of 15 μg) of the total number of HSV-1 genomes present in a single TG.

### Statistics

Analysis of numerical data was performed with the software packages Microsoft Excel and Modstat (Modern Microcomputers, Mechanicsville, VA). All viral titers were transformed by adding a value of 1 to the number of plaque-forming units detected per sample, such that all data could be analyzed on a logarithmic scale. The significance of differences between multiple groups was evaluated by one-way analysis of variance followed by a post hoc t-test. The significance of differences between strain 129 mice, *rag2*^-/- ^mice, *stat1*^-/- ^mice, versus *rag2*^-/- ^*stat1*^-/- ^mice was evaluated by two-way analysis of variance. The goodness of fit of the standard curve used in the competitive PCR assay was determined by regression analysis using the method of least squares.

## Abbreviations

GFP: green fluorescent protein

HSV: herpes simplex virus

ICP: infected cell protein

IFNAR: interferon-α/β receptor

IFNGR: interferon-γ receptor

LAT: latency-associated transcript

L/ST: long-short spanning transcript

p.i.: post inoculation

PML: promyelocytic leukemia

PKR: protein kinase R

RAG2: recombination-activated gene 2

R_L_: long-repeated

SCID: severe-combined immunodeficiency

Stat 1: signal transducer and activator of transcription 1

TG: trigeminal ganglia

## Competing interests

The author(s) declare that they have no competing interests.

## Authors' contributions

WPH conceived of the study and wrote the manuscript. WPH, DJJC, and BMG carried out pilot experiments in *scid *mice, NOD *scid *mice, PML^-/- ^mice, and *stat1*^-/- ^mice. WPH and JG carried out most of the *in vivo *experiments with *stat1*^-/- ^mice, *ifngr*^-/- ^mice, *rag2*^-/- ^mice, *stat1*^-/- ^mice, *rag2*^-/- ^*stat1*^-/- ^mice, *ifnar*^-/- ^mice, *ifnar*^-/- ^*ifngr*^-/- ^mice, and *rag2*^-/- ^*ifnar*^-/- ^mice. MS made and characterized the ICP0^- ^virus, 0^-^-GFP. JB constructed and characterized the wild-type virus, KOS-GFP. TPS and YI performed double labeling of trigeminal ganglia sections via fluorescent *in situ *hybridization for LAT RNA^+ ^neurons and antibody staining of viral antigen^+ ^neurons. CW performed *in vitro *comparisons of growth curves of ICP0^- ^null and ICP4^- ^null viruses in the presence or absence of interferon-β.
